# Mutasynthetic Production and Antimicrobial Characterization of Darobactin Analogs

**DOI:** 10.1128/spectrum.01535-21

**Published:** 2021-12-22

**Authors:** Nils Böhringer, Robert Green, Yang Liu, Ute Mettal, Michael Marner, Seyed Majed Modaresi, Roman P. Jakob, Zerlina G. Wuisan, Timm Maier, Akira Iinishi, Sebastian Hiller, Kim Lewis, Till F. Schäberle

**Affiliations:** a Justus-Liebig-University Gießen, Gießen, Germany; b German Center of Infection Research (DZIF), Partner Site Gießen-Marburg-Langen, Gießen, Germany; c Antimicrobial Discovery Center, Department of Biology, Northeastern University, Boston, Massachusetts, USA; d Fraunhofer Institute for Molecular Biology and Applied Ecology, Branch for Bioresources, Gießen, Germany; e Biozentrum, University of Basel, Basel, Switzerland; Georgia Institute of Technology

**Keywords:** Gram-negative antibiotics, BamA, RiPP reprogramming, natural products, heterologous gene expression

## Abstract

There is great need for therapeutics against multidrug-resistant, Gram-negative bacterial pathogens. Recently, darobactin A, a novel bicyclic heptapeptide that selectively kills Gram-negative bacteria by targeting the outer membrane protein BamA, was discovered. Its efficacy was proven in animal infection models of Escherichia coli, Klebsiella pneumoniae, and Pseudomonas aeruginosa, thus promoting darobactin A as a promising lead compound. Originally discovered from members of the nematode-symbiotic genus *Photorhabdus*, the biosynthetic gene cluster (BGC) encoding the synthesis of darobactin A can also be found in other members of the class *Gammaproteobacteria*. Therein, the precursor peptides DarB to -F, which differ in their core sequence from darobactin A, were identified *in silico*. Even though production of these analogs was not observed in the putative producer strains, we were able to generate them by mutasynthetic derivatization of a heterologous expression system. The analogs generated were isolated and tested for their bioactivity. The most potent compound, darobactin B, was used for cocrystallization with the target BamA, revealing a binding site identical to that of darobactin A. Despite its potency, darobactin B did not exhibit cytotoxicity, and it was slightly more active against Acinetobacter baumannii isolates than darobactin A. Furthermore, we evaluated the plasma protein binding of darobactin A and B, indicating their different pharmacokinetic properties. This is the first report on new members of this new antibiotic class, which is likely to expand to several promising therapeutic candidates.

**IMPORTANCE** Therapeutic options to combat Gram-negative bacterial pathogens are dwindling with increasing antibiotic resistance. This study presents a proof of concept for the heterologous-expression approach to expand on the novel antibiotic class of darobactins and to generate analogs with different activities and pharmacokinetic properties. In combination with the structural data of the target BamA, this approach may contribute to structure-activity relationship (SAR) data to optimize inhibitors of this essential outer membrane protein of Gram-negative pathogens.

## INTRODUCTION

The spread of antibiotic resistance in bacterial pathogens is limiting therapeutic options to combat bacterial infections. In Europe, North America, and Australia, 2.4 million deaths are expected from antibiotic-resistant infections over the next 30 years (1). In addition, it is estimated that these infections will amass costs amounting to a macroeconomic damage of $3.5 billion per year in the United States alone (1). Often termed the antimicrobial resistance (AMR) crisis, this situation is identified by the WHO as a major threat to global human health. To monitor this threat, the WHO started curating and evaluating data from all member states to create a priority list of bacterial pathogens, ranked according to criteria like treatability, preventability, prevalence of resistance, and more (2). The top half of this priority list is almost entirely composed of Gram-negative pathogens, and only 4 of the 25 bacterial species listed are Gram positive. This list is led by carbapenem-resistant Acinetobacter baumannii, Pseudomonas aeruginosa, and members of the family *Enterobacteriaceae*. Horizontal gene transfer on small genetic elements, such as plasmids, is common among Gram-negative bacteria, facilitating the rapid spread of newly evolved resistance mechanisms. Furthermore, Gram-negative bacteria possess an outer membrane (OM), which vastly restricts the accessibility of targets for drugs with intracellular modes of action. A dried-up antibiotic development pipeline is aggravating this situation and drastically illustrates that new, resistance-breaking antibiotics active against these highly threatening pathogens need to be discovered and developed now (3).

One promising candidate for further development as an antibiotic for the treatment of infections caused by multidrug-resistant (MDR) Gram-negative pathogens is the recently discovered natural product darobactin A (DAR A). It is produced by several *Photorhabdus* strains and shows good and selective *in vitro* activity against multiple Gram-negative pathogens. Most interestingly, Gram-positive bacteria and certain beneficial Gram-negative members of the bacterial gut microflora, e.g., from the evolutionarily distinct *Bacteroidetes* phylum, were not affected by this metabolite (4). Additionally, testing the efficacy in several mouse septicemia models against Escherichia coli, Klebsiella pneumoniae, and P. aeruginosa revealed good *in vivo* efficacy.

DAR A is a ribosomally synthesized and posttranslationally modified peptide (RiPP). Its biosynthesis is encoded by a small (∼6.5 kb) biosynthetic gene cluster (BGC) that codes for the precursor DarA, three transport-related proteins (DarB, -C, and -D), and the radical *S*-adenosylmethionine (SAM) (RaS) enzyme DarE (4, 5). This BGC is widespread in the investigated *Photorhabdus* genus, and orthologue BGCs could be identified in more genera of the class *Gammaproteobacteria*, such as *Vibrio*, *Pseudoalteromonas*, and especially *Yersinia*. In addition to the described and well-characterized DAR A structure, the analogs DAR B to -E were initially reported, based on the identification of the respective precursor peptides with altered core amino acid sequences in the GenBank database (4).

To test whether the sequences observed *in silico* translate into manufacturable products with the given biosynthetic machinery and, if so, how their activity patterns compare to that of DAR A, the goal was to make these predicted compounds accessible. In this study, we present the mutasynthetic production and antimicrobial characterization of the analogs DAR B to F identified *in silico*.

## RESULTS

### *In silico* identification of putative darobactin analogs.

The BGC corresponding to the production of the RiPP DAR A comprises five genes (4). During biosynthesis, the heptapeptide core is cleaved from the precursor peptide DarA and the radical SAM (RaS) enzyme DarE catalyzes the formation of two fused macrocyclic ring systems that endow DAR A with its rigid scaffold. Recently, we could show by heterologous expression experiments that DarA and DarE are solely essential for DAR biosynthesis (5). Using the amino acid sequence of DarE as the query, homologues were discovered in the BLAST database. This enabled the identification of DAR BGCs in the genera *Yersinia*, *Vibrio*, and *Pseudoalteromonas* of the gammaproteobacteria clade (4). Comparison of the DarA precursor sequences in the identified BGCs revealed that the DAR A heptapeptide core sequence (W^1^-N^2^-W^3^-S^4^-K^5^-S^6^-F^7^) is overall dominant. However, a few genes were identified that encode precursor peptides with one or two amino acid substitutions within the heptapeptide core. Among those, the sequence predicted to encode DAR B (W^1^-N^2^-W^3^-T^4^-K^5^-R^6^-F^7^) particularly caught our attention, since it was a precursor peptide gene that was present in addition to a DAR A-encoding gene within multiple *Photorhabdus* strains. Precursor-encoding genes that should result in DAR C (W^1^-S^2^-W^3^-S^4^-R^5^-S^6^-F^7^), DAR D (W^1^-N^2^-W^3^-S^4^-R^5^-S^6^-F^7^), and DAR E (W^1^-S^2^-W^3^-S^4^-K^5^-S^6^-F^7^) instead of the Dar A precursor were identified in *Yersinia* strains. In addition, the sequence W^1^-K^2^-W^3^-S^4^-K^5^-N^6^-L^7^, which we termed DAR F, was discovered in Photorhabdus thracensis (formerly *temperata*) strain DSM15199. In this case, a DAR A precursor was also present, and alignment of the precursors from various *Photorhabdus* strains indicated the DAR F sequence to be related to the DAR B precursor (Table 1).

**TABLE 1:** Alignment of translated precursor peptide amino acid sequences from different bacterial genera and strains

Organism and strain^*a*^	Darobactin type	Sequence^*b*^
		* * * * * * * * * * * * * * * * * * * *** ** **** **** * *
P. khanii DSM3369	A	MHNTLNETVKTQEALNSLAASFKETELSITDKALNELSNKPKIPEITA WNWSKSF QEI
P. heterorhabditis VMG (1)	B	MQNIPIETCKDQELLNSLVTSFKGTELSITEKALDELANNTEIPEINA WNWTKRF PI
P. heterorhabditis VMG (2)	A	MQNILVETCKTQEALNSLAASFKETELSITEKALNELSSKPKIPEITA WNWSKSF QEI
P. thracensis DSM15199 (1)	(F)	MQKIPTETCKNQELLNYLVTSFKGTELSITKKRLDELVNKTDIPDMTT WKWSKNL PI
P. thracensis DSM15199 (2)	A	MHNTSNEIIKTQEALNSLAASFKETELSITDKALNELSNKPNIPEITA WNWSKSF QEI
		************* **** * ** * ** *************
Vibrio vulnificus	A	MIIVEKEKVSISEKLDALMSSFSEMNLELRKFDQEKVNSINIAPPITA WNWSKSF
Pseudoalteromonas luteoviolacea	A	MIVEAPKEKVSISEKLDALKSSFSNQTLNIANVDQARVDSISVAPPITA WNWSKSF EK
		* * * ** * ** * * **** ** **
Y. enterocolitica	A	MFTSNQSNERINNTHLMALKTKLESLEQSFKNNLFSINDHEIENLRRSNSNNQITA WNWSKSF TQQ
Y. enterocolitica	D	MYTSHQSDLNTNNGKLIALKTKLEALDESFENNSLHISYDEIEKIKNNSLKSKITA WNWSRSF AEE
Y. bercovieri	A	MYTSHHTDRKTSNSNLMALKAKLESLDQSFKSNLLSISDHEIENLKNNNFNNEITA WNWSKSF TQQ
Y. bercovieri	E	MYTSHHTDRKTSNSNLMALKAKLESLDQSFKSNLLSISDHEIENLKNNNFNNEITAWSWSKSFTQQ
Y. frederiksenii	A	MYTSHQPEKKTSNTNLIALRTKLESLEESFKNSGLSIDAQEIENLKNSESENKITA WNWSKSF TQQ
Y. rohdei	A	MYTSRNPDGEIIPSNIMALLTKLGSLDESFKNNALTINNNEIENLKNSEVNNKITA WNWSKSF TQQ
Y. aldovae	A	MYNSTSHQKSHSVNNATALRSKLLSLQESFKSIPIHININKIEDLINSKSNNKITA WNWSKSF SQD
Y. aldovae	D	MFTSNQSNERINNAHLMALKAKLESLDESFKNNTLHISDNEIEKIKSNTLRSKITA WNWSRSF AEE
Y. massiliensis	A	MSISFQHQRKNNDQNLLALKSKLQSLGESFSHHSLYISNSELDKIRNSLAKTKITA WNWSKSF TEN
Y. pseudotuberculosis	D	MNPSSQSTVEKNNVNLIKLKSKLQSLEESFKNNPLYITSNEIDEVKNNTLHTKITA WNWSRSF AED
Y. pseudotuberculosis	C	MNPSSQSVVEKSNVNLIKLKSKLKSLEESFKNNPLYITSNEIDEIKNNTLHSKITA WSWSRSF AED

a *P*., *Photorhabdus*; *Y*., *Yersinia*. Alignments were done seperately for Photorhabdus, Vibrio and Pseudoalteromonas, and Yersinia strains.

b The precursor sequence is given, and the core amino acid sequence of the heptapeptide is underlined. The DAR F precursor peptide sequence (gray shading) is displayed in the alignment; however, due to strong sequence aberration, it was not taken into account for the amino acid identity, which is indicated by the asterisks.

**FIG 1:**
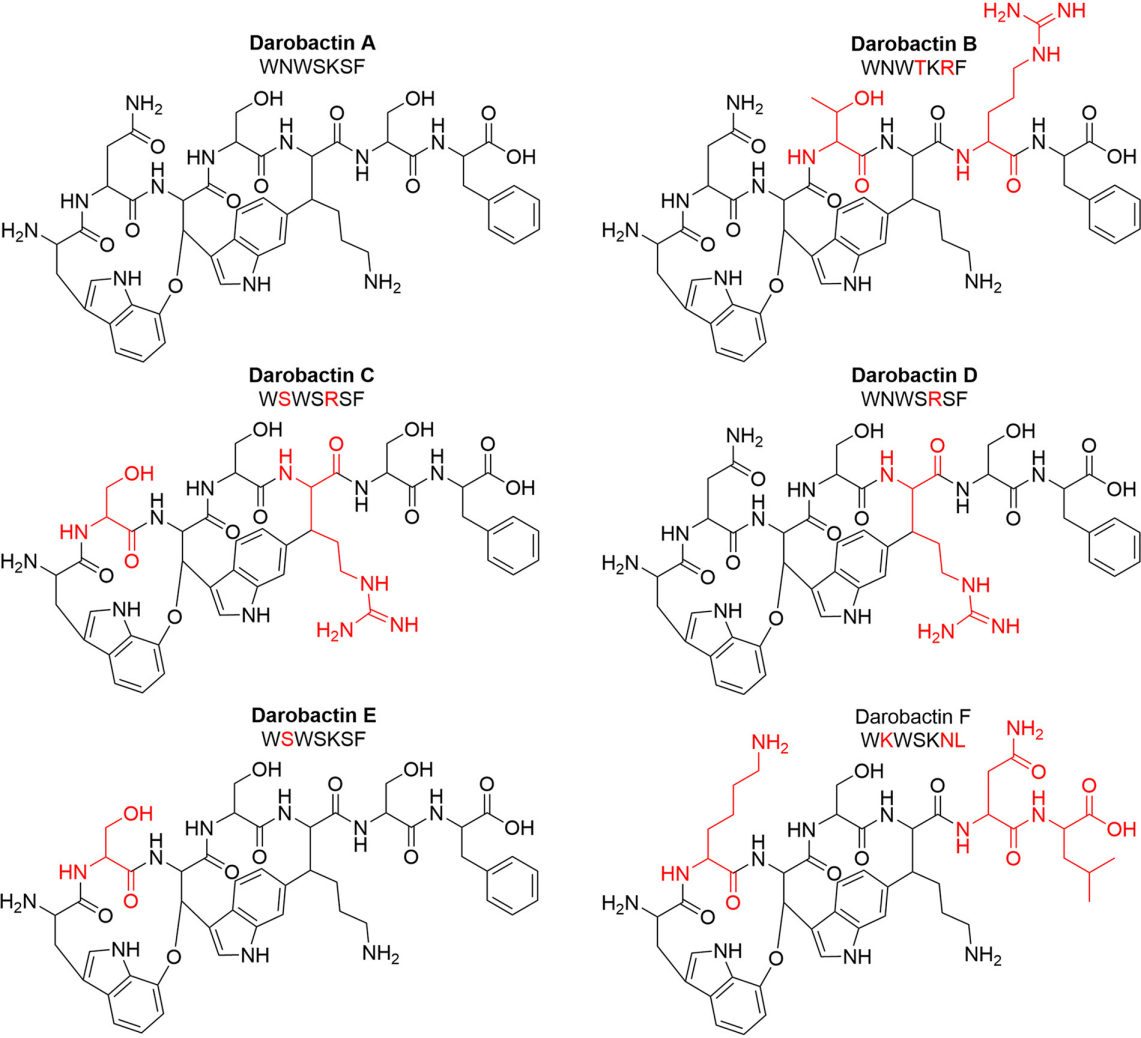
Structures of *in silico*-identified darobactin analogs. The amino acid sequences of the bicyclic heptapeptides are given. Predicted producer strains are of the genera *Photorhabdus* for DAR A, B, and F and *Yersinia* for DAR C, D, and E. The amino acids that differ from the DAR A sequence are highlighted in red. Exact masses and *m/z* values for [M + 2H]^2+^ (in brackets, as mostly the doubly charged ions were observed) for each of the structures are as follows: DAR A = 965.4032 Da (483.7089 *m/z*), DAR B = 1,048.4879 Da (525.2512 *m/z*), DAR C = 966.3984 Da (484.2065 *m/z*), DAR D = 993.4093 Da (497.7119 *m/z*), DAR E = 938.3923 Da (470.2034 *m/z*), and DAR F = 972.4818 Da (487.2482 *m/z*).

It was assumed that these precursors encode DAR analogs with an overall identical topology, since the *C-O-C* and *C-C* ring closures can be installed at identical (DAR B, E, and F) or very similar (DAR C and D) positions (Fig. 1). In a first approach to get hands on with these derivatives, five predicted producer strains, each carrying an alternative DAR precursor, were screened for activity in three different media at 1- and 20-fold concentrations. However, investigation of these extracts did not yield any congruent antibiotic hit in activity screenings, nor could any of the corresponding compounds be detected by mass spectrometry.

### Mutasynthetic production of darobactins B to F.

**FIG 2:**
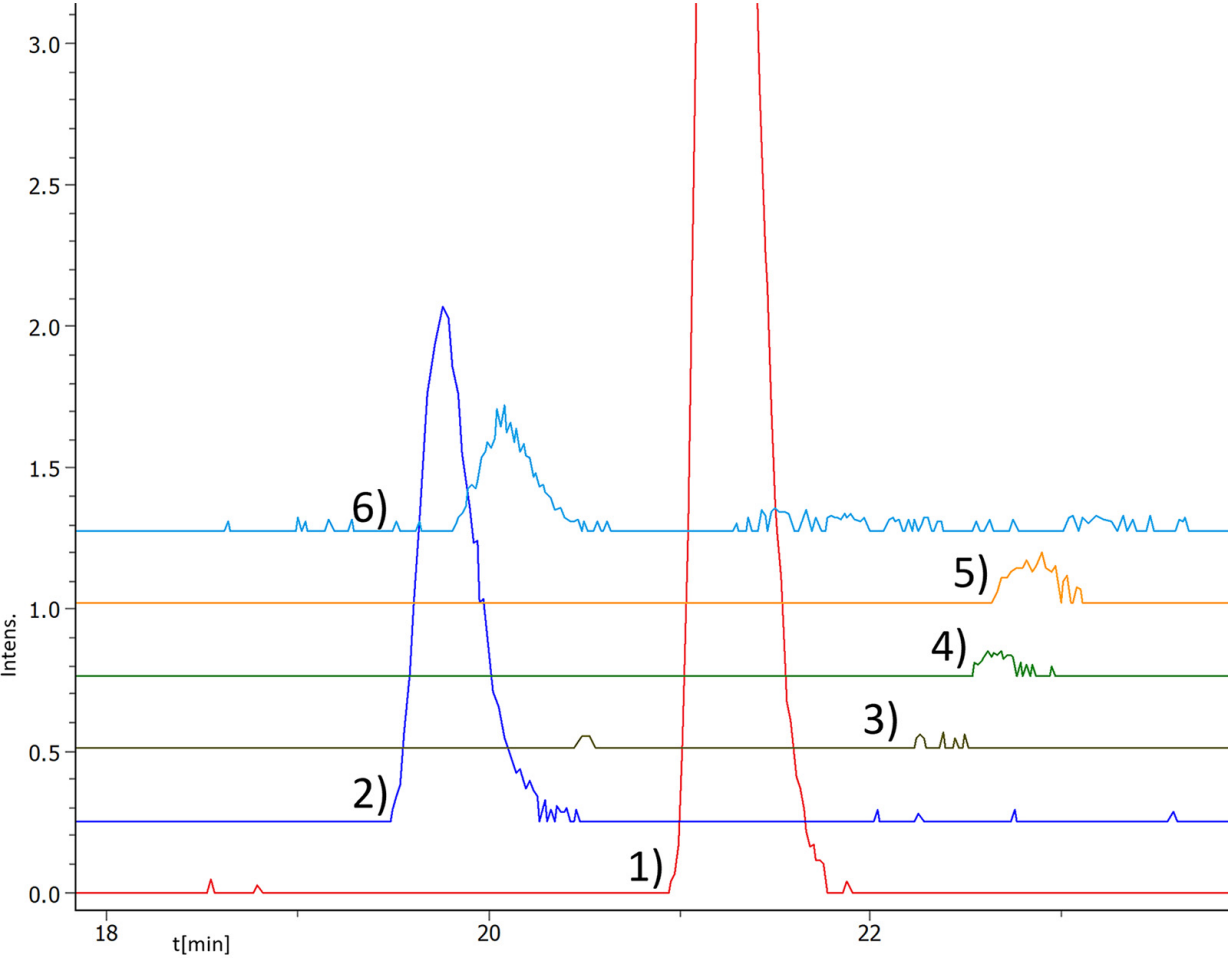
Extracted ion chromatograms (EICs, calcd. [M + 2H]^2+^ ± 0.01 Da) of darobactin analogs. Depicted are DAR B (trace 2, 525.2512 *m/z*), C (trace 3, 484.2065 *m/z*), D (trace 4, 497.7119 *m/z*), E (trace 5, 470.2034 *m/z*), and F (trace 6, 487.2482 *m/z*), with DAR A (trace 1, 483.7089 *m/z*) as the control, showing the creation of these derivatives by mutasynthesis. Chromatogram traces have an offset. Complete chromatograms are given in Fig. S2.

To get access to the DAR analogs identified *in silico*, the original heterologous expression system based on the Photorhabdus khanii DSM3369 BGC was modified. The *darA* sequence encoding the core amino acids was exchanged against a *lacZ* spacer derived from pCRISPOMYCES-2 (6), and Goldengate assembly was used to incorporate the sequences of the desired core amino acids (Fig. S1 in the supplemental material). However, exchanging the core amino acids of DarA while maintaining the Q-E-I follower did not result in detectable formation of DAR analogues. Therefore, we tested whether the three follower amino acids Q-E-I are essential for the heterologous production of DAR A in this system. Removal of these amino acids resulted in a comparable production yield (Fig. S2), as was also observed for a different expression vector system (5). Hence, the follower peptide was omitted in the following constructs and chimeric precursors without the follower peptide were generated, consisting of the P. khanii DSM3369 leader and the sequences of DAR B to F, respectively. Following heterologous expression in E. coli cells, the expected mass-to-charge ratios of the corresponding *in silico*-predicted natural derivatives were detected by high-resolution ultra-high-performance liquid chromatography–mass spectrometry (HR-UHPLC-MS) analysis. The identity of the molecules was corroborated by tandem mass spectrometry (MS/MS) fragmentation analysis. In general, the OH-b_1_* and OH-b_2_* fragments, indicative of hydroxylated tryptophan (W^1^; cleavage of the *C-O-C* ether bridge), as well as the C-terminal fragments y_1_ and y_2_, could be observed (Fig. S3). DAR C alone could not be fragmented, due to a poor production titer. The retention times were similar to that of DAR A, with DAR B and F eluting slightly earlier. However, the intensities of the ions corresponding to the desired molecules showed a high degree of variation (Fig. 2). Due to the similarity of the derivative ions, the peak intensity could be approximately correlated with the expression level. The second highest expression level following that of DAR A was observed for DAR B (*m/z *= 525.2533 [M + 2H]^+^, Δppm = 4) (Fig. 2). DAR F and E showed intensities of <10% compared to the DAR A peak. DAR C and D (carrying the K^5^R exchange [a change of K to R at position 5], one of the three amino acids undergoing posttranslational modification) were only detected in traces, with DAR C showing the lowest intensity of all (Fig. 2). On the other hand, the production yield observed for DAR F was in the same range as for DAR B, thereby showing that even multiple modifications are possible without impairing the production drastically.

After corroboration of the identity of the molecules by MS/MS fragmentation analysis, the BGCs were recloned into the optimized heterologous expression system (5) to reach higher production titers. However, even using this system, DAR C could not be obtained due to the poor production yield, and only ∼1 mg of DAR D and E was isolated to enable the initial MIC testing. The DAR B expression yield was >30-fold higher, which enabled subsequent one- and two-dimensional (2-D) nuclear magnetic resonance (NMR) experiments.

**FIG 3:**
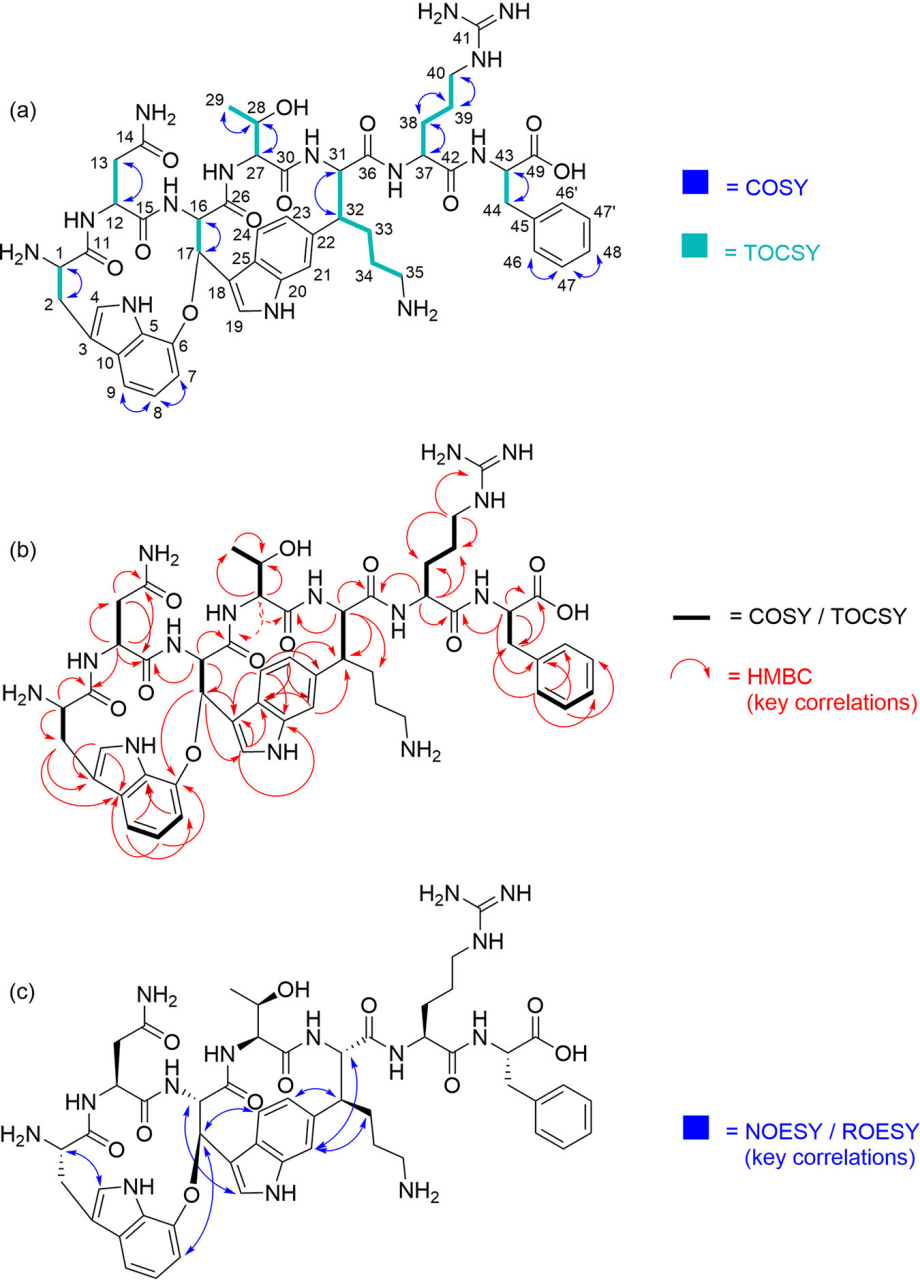
(a) Correlation spectroscopy (COSY) and total correlation spectroscopy (TOCSY) correlations, (b) key heteronuclear multiple-bond coherence (HMBC) correlations, and (c) key nuclear Overhauser effect spectroscopy (NOESY) and rotating-frame nuclear Overhauser effect spectroscopy (ROESY) correlations of darobactin B.

The ^1^H and ^13^C NMR spectra of DAR B in D_2_O showed seven C_α_ methine signals for the peptide backbone (δ_H_, 4.77, 4.74, 4.34, 4.22, 4.08, 3.77, and 3.37 ppm; δ_C_, 67.81, 64.51, 62.52, 59.11, 58.52, 57.81, and 55.19 ppm). The amino acid sequence could be determined by a thorough 2-D NMR analysis to be Trp^1^-Asn-Trp^2^-Thr-Lys-Arg-Phe. Heteronuclear multiple-bond coherence (HMBC) correlations further confirmed ring closure between C_β_ of Trp^2^ (δ_H_ = 6.24 ppm) and the aromatic ring of Trp^1^ (δ_C_ = 149.59 ppm) via a bridging oxygen atom, as well as the *C*-*C* bond between C_β_ of lysine (δ_C_ = 52.40 ppm) and the aromatic ring of Trp^2^ (δ_H_ = 6.99 ppm). Characteristic ^1^H signals in this respect are the doublet of doublets at 6.99 ppm for Trp 23 and the doublet at 6.24 ppm for Trp 17. In addition, the ^13^C shift of Lys 32 at 52.40 ppm is quite typical for the DAR derivatives. Due to the proximity of the observed heteronuclear single quantum coherence (HSQC) correlations, assignment of the proton signals for the lysine residue proved difficult. On the other hand, the ^13^C signal of Lys 33 was identified by its HMBC coupling with the proton of Lys 31, while the ^13^C signal of Lys 35 was mainly assigned based on its chemical shift (and a comparison with other DAR derivatives, as well as a spectral simulation) (Fig. 3). Distinction of the proton signals for Lys 33 and Lys 34 was based on the observed rotating-frame nuclear Overhauser effect spectroscopy (ROESY) correlation between Lys 33 and Trp 21, which is in agreement with the data reported for DAR A (4). The ^13^C NMR spectrum additionally indicates the presence of trifluoroacetic acid (TFA), which is probably derived from the purification procedure and might form a salt with the alkaline amino acid residues present in the molecule. The key nuclear Overhauser effect spectroscopy (NOESY) and ROESY correlations are in accordance with those published for DAR A (4).

### Bioactivity of darobactin analogs.

The isolated DAR B, D, and E were tested against a panel of clinically relevant Gram-negative pathogens, including drug-resistant isolates. DAR F, which was inactive in a preliminary agar diffusion test using concentrated supernatant from the expression culture, was not pursued further. DAR D and E, which were generated based on the amino acid sequences detected in the *Yersinia* genus, had 8- to 16-fold-higher MIC values than DAR A. DAR B displayed comparable or even better activities (Table 2). In a next step, DAR B was further evaluated in comparison to DAR A by using an expanded panel of test organisms (Table 3). Both compounds selectively kill Gram-negative pathogens but do not inhibit the growth of commensal gut bacteria, such as Bacteroides fragilis. E. coli strains with an altered *bamA* sequence showed resistance to both derivatives. Peculiarly, we observed DAR B to be slightly more potent against A. baumannii strains. Therefore, additional clinical isolates of A. baumannii were tested. In this panel, DAR B maintained its 2-fold-increased activity against all clinical isolates of A. baumannii, including five MDR strains. Additionally, cytotoxicity assays were performed using HepG2 human liver cancer, Hek293 human embryonic kidney, and human epithelial FaDu cells. No activity was detected for any of the tested cell lines up to 128 μg/ml, indicating low cytotoxicity.

**TABLE 2:** MICs of darobactin A, B, D, and E against Gram-negative pathogens

Organism and strain	MIC (μg/ml) of:			
	DAR A	DAR B	DAR D	DAR E
Klebsiella pneumoniae DSM30104	4	1–2	64	32
Salmonella enterica ATCC 13076	8	1	64	32
Escherichia coli ATCC 35218	4–8	1	64	32
E. coli NRZ14408	4	0.5–1	64	32
E. coli K0416	4	0.5–1	32	32
E. coli Survcare052	4–8	1	>64	64
E. coli MMGI1	4	1	64	32
Acinetobacter baumannii ATCC 19606	>64	32	>64	>64

**TABLE 3:** MICs of darobactin A and B against pathogens, a gut commensal, darobactin-resistant strains, and human cell lines

Organism and strain or cell line	MIC (μg/ml) of:	
	DAR A	DAR B
Pathogens		
Klebsiella pneumoniae ATCC 700603	2	2
Salmonella enterica serovar Typhimurium LT2 ATCC 19585^*a*^	2	2
Escherichia coli ATCC 25922	2	2
E. coli MG1655	4	8
E. coli AR350 (*mcr-1*)	2	4
Pseudomonas aeruginosa PAO1	2	2
P. aeruginosa PA14	16	8
Acinetobacter baumannii ATCC 17978	8	4
A. baumannii LAC-4	8	4
A. baumannii AB5075-UW	32	8
A. baumannii AB307-0294	32	16
A. baumannii ACICU	64	32
A. baumannii AB057	64	32
A. baumannii AYE	128	64
Moraxella catarrhalis ATCC 25238	8	8
Enterobacter cloacae ATCC 13047	32	128
Proteus mirabilis ATCC 7002	64	128
Stenotrophomonas maltophilia ATCC 13637	>128	>128
Staphylococcus aureus HG003	>128	>128

Gram-negative gut commensal		
Bacteroides fragilis ATCC 25285^*a*^	>128	>128

Darobactin-resistant E. coli strains (*bamA* mutants)		
E. coli MG1655 *bamA* mutant strain 1	>128	>128
E. coli MG1655 *bamA* mutant strain 2	>128	>128
E. coli MG1655 *bamA* mutant strain 3	>128	>128

Human cell lines		
FaDu	>128	>128
Hek293	>128	>128
HepG2	>128	>128

a Grown under anaerobic conditions.

### Binding to the target BamA.

With the recent cocrystallization of BamA and DAR A (7), it was clearly shown that the inhibition of BamA’s function relies on the interaction of the peptidic backbone with the β-barrel domain. Thereby, about one third of the DAR A surface is in contact with BamA, the membrane, and the aqueous surrounding, respectively. A similar mode of action could be expected for DAR B, based upon the similar activity data even against DAR A-resistant E. coli strains (Table 3). If it is the case that both compounds bind to the same site, there should be no synergistic effect of bacterial killing when delivered in combination. To test this, checkerboard assays against E. coli MG1655 and K. pneumoniae ATCC 700603 were performed. The fractional inhibitory concentration (FIC)index mean values were 1.09 for both strains, indicating an additive effect (8).

**FIG 4:**
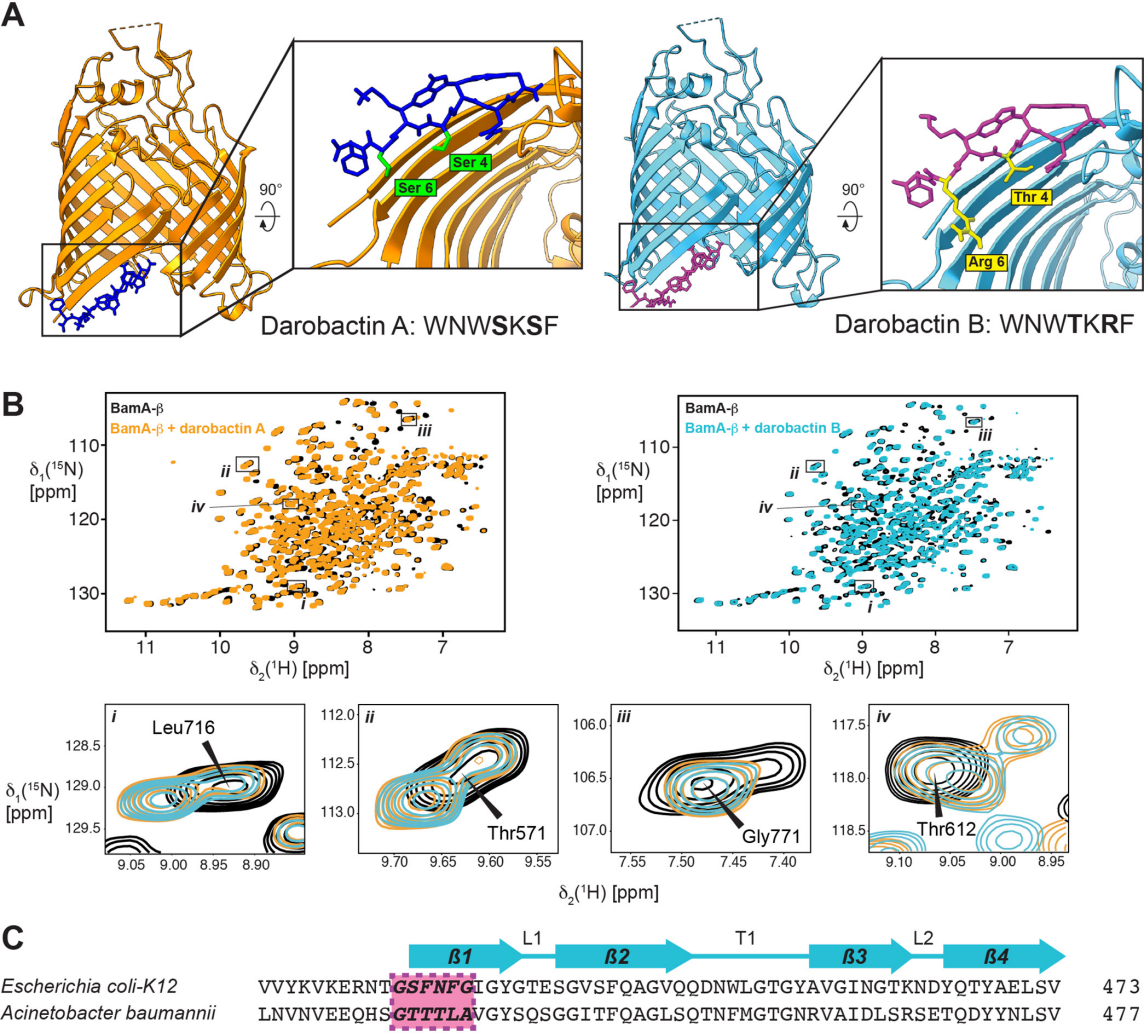
Structural analysis of darobactin B binding to BamA-β. (A) Comparison of the X-ray structures of BamA-β with bound DAR A (orange/dark blue) and BamA-β with bound DAR B (light blue/magenta). (B) Two-dimensional [^15^N,^1^H] transverse relaxation-optimized spectroscopy–heteronuclear single quantum coherence (2D [^15^N,^1^H]-TROSY-HSQC) spectra of apo-BamA-β (black) and BamA-β after the addition of 1 equivalent of either DAR A (orange) or DAR B (light blue). Selected examples of resonance peaks with their assignments given are shown as closeup figures below. (C) Sequence alignment of segments of the BamAs from E. coli and A. baumannii. The darobactin binding site is shown in purple. The locations of secondary structure elements are indicated above the alignment.

To confirm that both compounds compete for the same binding site, we used solution NMR spectroscopy and X-ray crystallography experiments (Fig. 4). The high-resolution crystal structure of DAR B, resolved at 2.4 Å, revealed that DAR B binds to the same binding site in E. coli BamA as was identified for DAR A (Fig. 4A) (7). Thereby, the amino acid side chains of residues T^4^ and R^6^ in DAR B are pointed toward the barrel lumen. Furthermore, the binding behavior was also compared in aqueous solution by NMR experiments. The 2D [^15^N, ^1^H] transverse relaxation-optimized spectroscopy (TROSY)-HSQC NMR spectra of the transmembrane β-barrel domain of BamA bound to either DAR A or DAR B superimposed essentially perfectly, indicating that the two ligands bind in identical fashion and cause the same structural rearrangements of BamA (Fig. 4B). Notably, the amino acid composition of the binding site on strand 1 is not conserved between E. coli and A. baumannii (Fig. 4C), which is in agreement with the backbone-dominated interaction mode and the variability of side chains 4 and 6 between DAR A and B.

### Plasma protein binding.

**FIG 5:**
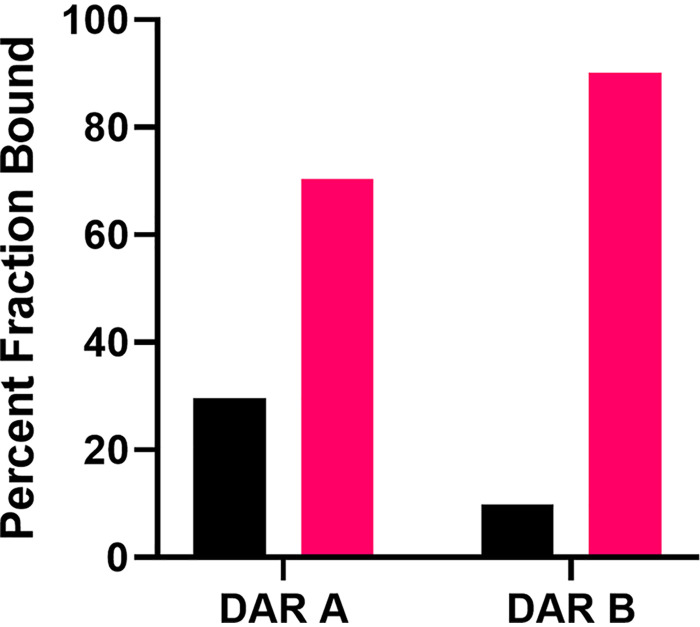
*In vitro* binding of darobactin A and B to plasma protein. The percentages of DAR A and DAR B that either bound to human plasma protein (pink bar) or were unbound in the buffer (black bar) are given.

DAR A has good exposure in mouse models of infection, with blood levels maintained above the MIC for 8 h and a half-life of 1 h (4). To gain insight into whether the amino acid variance of DAR B would alter the pharmacokinetic properties, plasma protein binding was investigated (Fig. 5). The fractions of the respective compounds bound and unbound to plasma proteins were 70.4% and 29.6% for DAR A and 90.1% and 9.9% for DAR B.

## DISCUSSION

The natural product DAR A was initially discovered by screening bacterial strains of the genus *Photorhabdus*, which are known symbionts of entomopathogenic helminths that feed on insect larvae (4). The symbiotic bacteria produce specialized metabolites, i.e., neurotoxins, to immobilize the prey and biosynthesize antibacterial compounds to protect the food source from other microorganisms (9, 10). It can be hypothesized that like other RiPPs (e.g., microcins), the antibiotics of the DAR class are effective metabolites used in an ecological context to suppress immediate Gram-negative competitors, thus shaping the microbiome they are part of (11). *Photorhabdus* strains colonize the gut of the nematode, like other *Enterobacteriaceae* (e.g., E. coli), and are released by the nematode. The latter mechanism will also release potential competitors, which are then inhibited by DAR. Even though the natural product was first identified from *Photorhabdus* strains, the corresponding BGC is more widespread among gammaproteobacteria. All of these (e.g., *Pseudoalteromonas*, *Vibrio*, and *Yersinia*) are reported to be associated with higher organisms (12–14).

Interestingly, some of the *Photorhabdus* strains were found to carry a second precursor peptide with an altered core and follower amino acid sequence, already hinting toward more molecules of the DAR class to be discovered. Furthermore, some of the *in silico*-identified precursors in orthologous clusters in *Yersinia* strains also showed altered core amino acid sequences (of DAR B and DAR E), despite being the only precursor in the respective BGC. The predicted derivatives were not directly accessible by fermentation approaches. However, heterologous expression opens up the possibility to biosynthesize specialized metabolites, even though the BGC is silent under laboratory conditions. Previously reported DAR A expression constructs were successfully modified to generate the desired analogs. However, DAR B alone was isolated in a scale of several mg, while heterologous expression of DAR D and E resulted in ∼1 mg of pure compound from ∼100 liters of fermentation broth, and DAR C was not isolated at all. The identity of the molecules could be confirmed by MS/MS fragmentation analysis and was verified by NMR experiments in the case of DAR B. However, this synthetic biology approach does not clarify whether these analogs are real natural products or if these sequences only represent snap shots in evolution that will be deselected, since they are not easy to biosynthesize. It also might be that the modifying DarE is not adapted to the altered sequence of the core heptapeptide yet, so that much of the precursor is not converted into the final bicyclic compound. It can be assumed that it will depend on the bioactivity of the analog whether it is worthwhile from an evolutionary point of view to keep the sequence and to optimize biosynthesis of the metabolite. For DAR C and E, the K-to-R shift at position 5, an amino acid that participates in the aromatic-aliphatic covalent *C-C* bond, resulted in severely impeded production compared to the production of DAR A. Further investigation of DarE’s flexibility will be part of future research.

Concerning their mode of action, the DARs bind to the promising target BamA, which is located in the OM of Gram-negative bacteria and not yet employed for any antibiotics in application. The MICs observed for DAR A and B proved the compounds to possess activity against MDR pathogens. Clinical isolates resistant to rifampicin (E. coli strain NRZ14408), tetracycline (E. coli strains NRZ14408, MMGI1, and K0416), gentamicin (E. coli strains NRZ14408 and MMGI1) and last-resort carbapenems (E. coli strains NRZ14408, K0416, and Survcare052) showed MICs similar to those of wild-type E. coli. According to the 2019 CDC report on antimicrobial resistance, carbapenem-resistant *Enterobacteriaceae* (CRE), and carbapenem-resistant Acinetobacter in particular, are “urgent threats” to the modern health care system (15). Treatment options are needed, since prevention measures are not enough; e.g., during the COVID-19 pandemic, an outbreak of NDM-1 producing Klebsiella pneumoniae in an intensive care unit in Paris (March 2021) could not be prevented, although strict additional infection control procedures were applied.

New antibiotics, such as darobactin, with potency against these pathogens are desperately needed. In contrast, DAR D and E had significantly lower activities against the whole panel of test strains, with 8- to 16-fold-elevated MICs compared to those of DAR A. Hence, either these analogs target as-yet-unidentified bacteria or they indicate a degeneration process of the BGC in these strains. DAR F, which is part of a second precursor in addition to DAR A, points in this direction, since this bicyclic heptapeptide showed no activity. This can be explained by the fact that this is the only derivative investigated not ending with the amino acid phenylalanine, which represents the most frequent amino acid of known and putative β signals from bacterial OM proteins (7). Therefore, DAR F would not block the β signal binding site and, thus, would not inhibit the function of BamA. The N^2^S exchange, present in DAR C, E, and F, results in 8- to 16-fold decreases in the potency of the tested DAR E compared to that of DAR A against various E. coli strains. Since Ser2 should eliminate the side chain interactions existing between Asn2 and Lys808 and Asn427 of the BamA β1 strand, it is likely that this greatly reduces DAR E’s affinity for the target and, thus, reduces its potency. In contrast to DAR A, DAR D carries an arginine in position 5 instead of lysine. If DAR A is bound to BamA, the Lys5 side chain points away from BamA, thereby enabling interaction with the negatively charged phosphate groups of phosphatidylglycerol and cardiolipin lipids (7). Supposedly, arginine could act similarly in the place of lysine, as arginine attracts more phosphate than lysine in membranes and can form more hydrogen bonds with phosphate clusters than lysine (16). Arginine is also capable of cardiolipin binding, a further important aspect of DAR and BamA binding (17). Therefore, the 16-fold-lower potency of DAR D was unexpected, and further molecular docking studies of DAR analogs and BamA in combination with experimental data will likely provide evidence as to why this derivative has reduced activity.

DAR B shares a similar spectrum of activity with DAR A, with a 2-fold increase in activity against A. baumannii, which was observed across multiple strains in both wild-type and clinical isolates. Carbapenem-resistant A. baumannii is currently one of the most threatening bacterial pathogens, with certain infections reaching 60% mortality (18–21). Even though the MICs for the clinical isolates varied by 14-fold, they were consistent between DAR A and DAR B. It appears that certain resistance mechanisms in MDR isolates may apply to DARs, but not in all cases, such as the hypervirulent and MDR A. baumannii clinical isolate LAC-4, which as we show has MICs of 8 μg/ml and 4 μg/ml for DAR A and B, respectively. Comparing the resistance determinants between the DAR-susceptible strain LAC-4 and the DAR-resistant A. baumannii strain AYE, an explanation may lie in A. baumannii’s unique colistin resistance mechanism of removing the lipid A-containing lipopolysaccharides (LPSs). Lipid A biosynthesis is essential for most Gram-negative bacteria, but certain colistin-resistant strains of A. baumannii, including AYE, are capable of survival without lipid A (22). These changes in the OMs’ compositions will alter their properties. In addition, *Moraxella* strains that are less affected by DARs than E. coli also possess another OM structure, e.g., fewer or no LPSs and fewer OM proteins. Whether such less-complex OMs may reduce the need of a functional β-barrel assembly machinery and thereby affect the potency of DAR should be further tested in future.

We could demonstrate that DAR B binds more to plasma proteins than DAR A, suggesting a longer elimination half-life and smaller amount of the free compound in plasma. These pharmacokinetic properties may be an advantage where longer times of exposure to antibiotics are required to clear infections and/or where frequent dosing is disadvantageous.

### Conclusion.

The DAR class is inevitably going to expand with the increase in available genome data, since a multitude of related precursor peptides and RiPP BGCs can be identified *in silico*. Based on the experience from this project, this could become a compound class in which discovery is driven by targeted bioinformatics-guided approaches, followed by subsequent expression in cell-based production systems, rather than by classical screening approaches. This will enhance the probability of success for future development of BamA inhibitors with activity against the most critical Gram-negative priority pathogens.

## MATERIALS AND METHODS

### Darobactin analog screening of predicted producers.

Strains including Yersinia frederiksenii ATCC 33641, Photorhabdus asymbiotica (KLE collection), Yersinia pseudotuberculosis ATCC 6904, Yersinia aldovae ATCC 35236, and Yersinia bercovieri ATCC 43970 for DAR analogs A, C, D, and E, respectively, were grown in 10-ml fermentations for 10 days at 28°C and 220 rpm. Aliquots were taken at time points of days 3, 7, and 10. Twenty-fold-concentrated supernatants were dried by rotary evaporator and resuspended in MilliQ water. Five-microliter amounts of 1× supernatant and 20× concentrated supernatant were pipetted onto Mueller-Hinton II agar lawns with E. coli strain MG1655 and observed for zones of inhibition.

### Mutasynthetic production of darobactin analogs.

E. coli strains for cloning and expression were grown in LB broth or on agar medium supplemented with appropriate antibiotics or supplements at 37°C or 30°C using standard working concentrations. Plasmid DNA was isolated using the innuPREP plasmid minikit 2.0 (AnalytikJena, Jena, Germany) according to the manufacturer’s protocol. Genomic DNA was extracted using the innuPREP bacteria DNA kit (AnalytikJena, Jena, Germany). PCR amplification for cloning purposes was performed using Q5 DNA polymerase (NEB Biolabs, USA) according to the manufacturer’s instructions. Restriction digestion was performed using standard techniques and employing NEB enzymes (NEB Biolabs, USA). DNA fragments were analyzed on and excised from 1% or 2% Tris-acetate-EDTA (TAE)-agarose with GeneRuler 1 kb plus (ThermoFisher, Waltham, USA) as the marker. DNA for cloning purposes was purified using the Zymoclean large-fragment DNA recovery kit according to the manufacturer’s instructions. DNA concentrations were determined photometrically with an Eppendorf BioSpectrometer (Eppendorf AG, Hamburg, Germany), using a 1-mm-light-path UV cuvette. DNA fragments to be fused by isothermal assembly were gel purified and fused using self-made isothermal assembly master mix (23) using NEB enzymes (NEB Biolabs, New Brunswick, USA). The assembled plasmids were transferred to E. coli cells using standard electroporation protocols (4, 5).

Construction of pNBDaroMod for modification of the precursor peptide was performed by simultaneously linearizing pNB03 (4) by PCR using 5′-TCCCTTAACGTGAGTTTTCG-3′/5′-TTTTATAACCTCCTTAGAGCTCGAA-3′, amplifying truncated *darA* (50 nt missing from the 3′-terminus) using 5′-GCTCTAAGGAGGTTATAAAAATGCATAATACCTTAAATGAAACCGTTAAA-3′/5′-TAGGTTTATTGCTTAATTCGTTTAGTGCTT-3′, amplifying the lacZ spacer from pCRISPOMYCES-2 using 5′-CGAATTAAGCAATAAACCTAAAGTCTTCTCAGCCGCTACA-3′/5′-ACCTGATGGGATAAGCTTTAATGTCTTCACCGGTGGAAAG-3′, amplifying the rest of the *P. khanii* DSM3369 BGC using 5′-TAAAGCTTATCCCATCAGGTTATTT-3′/5′-CGAAAACTCACGTTAAGGGATTACGCCGCGATGGTTTGTTTTATT-3′, and subsequent isothermal assembly of the fragments to yield the final plasmid. After transformation and selection on LB with kanamycin, apramycin, IPTG (isopropyl-β-d-thiogalactopyranoside), and X-Gal (5-bromo-4-chloro-3-indolyl-β-d-galactopyranoside) (LB_Kan/Apra/IPTG/X-Gal_), blue colonies were picked and the correct assembly of the plasmid was corroborated by test restriction. Amino acid modifications were designed *in silico* and ordered as complementary oligonucleotides (Table S1) with 4-nucleotide overlaps of the pNBDaroMod backbone. Oligonucleotides were annealed and assembled into pNBDaroMod using the protocol described in Cobb et al. (6), and the resulting plasmids were transferred to E. coli BW25113 and selected on LB_Kan/Apra/IPTG/X-gal_. White colonies were picked and grown in LB_Kan/IPTG_ for 3 days at 220 rpm and 30°C. The correct assembly of the plasmid was corroborated by UHPLC-MS profiling, i.e., detection of the expected product ion. For increased production titers, the modified BGCs were recloned into pRSF-duett, using the primers 5′-GTATAAGAAGGAGATATACAATGCATAATACCTTAAATGA-3′/5′-TGCTCAGCGGTGGCAGCAGCTTACGCCGCGATGGTTTGTT-3′ in order for all constructs to match the layout of pRSF-ADC5, and produced in E. coli strain BAP1 (5).

Isolation of compounds and structure elucidation are described in the supplemental material.

### MIC testing and checkerboard assays.

The MICs were determined by broth microdilution assays in round-bottom 96-well plates. For details, see the supplemental material.

### Crystallization, X-ray data collection, and structure determination.

Samples of the BamA β-barrel (BamA-β) for crystallization were produced as described previously (7). BamA-β was crystallized from buffer (10 mg/ml in 20 mM Tris, pH 7.5, 150 mM NaCl, 0.35% C8E4 [tetraethylene glycol monooctyl ether], 0.05% LDAO [*N*,*N*-dimethyldodecylamine *N*-oxide]) with a 2-fold excess of DAR B in a sitting-drop vapor diffusion experimental setup. Crystals in space group I2 were grown in 0.2 M calcium chloride dihydrate, 0.1 M HEPES, pH 7.5, 53% (vol/vol) polyethylene glycol 400 (PEG 400) and directly flash frozen in liquid nitrogen. Data were collected at the SLS beamline X06DA (Swiss Light Source, Paul Scherrer Institute, Switzerland) at 100 K, indexed and integrated with XDS (24), and scaled using Aimless (25). The structure was solved by molecular replacement using the crystal structure with PDB code 7NRF (7) as the search model, using the program Phaser (26). Model building was performed with Coot (27), ligand restraints were generated with eLBOW (28), and the model was refined in PHENIX (29). MolProbity (30) was used to evaluate the final model, and Chimerax (31) for protein model visualization. Data and refinement statistics are summarized in Table S4. The atomic coordinates have been deposited in the RCSB Protein Data Bank and are available under the accession code 7P1C.

### Solution NMR spectroscopy for binding mode studies.

[*U*-^15^N,^2^H]-labeled BamA-β for solution NMR studies was expressed and purified as described previously (32, 33). The protein was purified on a size exclusion column in 20 mM HEPES, 150 mM NaCl, 0.1% LDAO (wt/vol) at pH 7.5, and then samples were concentrated to a final protein concentration of 250 μM. 2-D [^15^N,^1^H]-TROSY-HSQC experiments were measured on a 700-MHz Bruker spectrometer equipped with a cryogenic probe at 37°C. One hundred twenty-eight transients were accumulated, with 1,024 and 256 complex points in the ^1^H and ^15^N dimensions, respectively. Titrations of BamA-β were done with 1.0 molar equivalent of either DAR A or DAR B. The data were processed using Topspin 3.6.2 (Bruker Biospin) and analyzed using NMR Sparky (34).

Plasma protein binding (35) was determined by using a rapid equilibrium dialysis (RED) plate (catalog number 90006; ThermoFisher), and MassHunter qualitative analysis (Agilent Technologies) and Skyline (MacCoss Lab Software, University of Washington) were used to perform relative quantification of DAR A and DAR B (36). For details, see the supplemental material.

### Data availability.

The atomic coordinates have been deposited in the RCSB Protein Data Bank and are available under accession code 7P1C.
