# Supplementary material for: Mutasynthetic Production and Antimicrobial Characterization of Darobactin Analogs
Source: Microbiol Spectr. 2021 Dec 22;9(3):e01535-21. doi: 10.1128/spectrum.01535-21 (PMC8694152; doi:10.1128/spectrum.01535-21)
Supplement: SUPPLEMENTAL FILE 1 — Supplemental material. Download SPECTRUM01535-21_Supp_1_seq10.pdf, PDF file, 6.3 MB [file spectrum01535-21_supp_1_seq10.pdf]

Supporting Information

Mutasynthetic production and antimicrobial characterisation of darobactin analogs

Nils Böhringer[1,2,+], Robert Green[3,+], Yang Liu[1], Ute Mettal[1], Michael Marner[4], Seyed Majed Modaresi[5], Roman P. Jakob[5], Zerlina G. Wuisan[1], Timm Maier[5], Akira Iinishi[3], Sebastian Hiller[5], Kim Lewis[3,*] and Till F. Schäberle[1,2,4,*]

[1] Justus-Liebig-University Gießen, 35392 Gießen, Germany; [2] German Center of Infection Research (DZIF), Partner Site Gießen-Marburg-Langen, 35392 Gießen, Germany; [3] Antimicrobial Discovery Center, Department of Biology, Northeastern University, Boston MA, USA; [4] Fraunhofer Institute for Molecular Biology and Applied Ecology (IME), Branch for Bioresources, 35392 Gießen, Germany; [5] Biozentrum, University of Basel, 4056 Basel, Switzerland.

+ Equal contribution

* Correspondence: k.lewis@northeastern.edu and till.f.schaeberle@agrar.uni-giessen.de

Content

Isolation of compounds 3

Structure elucidation 4

Minimum inhibitory concentration testing 4

Checkerboard Assays 5

Plasma Protein Binding 6

Figure S1: General cloning strategy for exchange of core AAs in the DAR A precursor 7

Figure S2A: Extracted ion chromatogram (EICs, calcd. [M + 2H+]2+ ± 0.01 Da) of DAR A 8

Figure S2B: Extracted ion chromatograms (EICs, calcd. [M + 2H+]2+ ± 0.01 Da) of DAR A (483.7089 m/z) and the DAR analogues 9

Figure S2C: Extracted ion chromatogram (EICs, calcd. [M + 2H+]2+ ± 0.01 Da) of DAR B 10

Figure S2D: Extracted ion chromatogram (EICs, calcd. [M + 2H+]2+ ± 0.01 Da) of DAR C 11

Figure S2E: Extracted ion chromatogram (EICs, calcd. [M + 2H+]2+ ± 0.01 Da) of DAR D......12
Figure S2F: Extracted ion chromatogram (EICs, calcd. [M + 2H+]2+ ± 0.01 Da) of DAR E 13
Figure S2G: Extracted ion chromatogram (EICs, calcd. [M + 2H+]2+ ± 0.01 Da) of DAR F......14
Figure S3A: Structure of the N-terminal W of Darobactin Analogues with plausible MS/MS fragmentation pattern 15
Figure S3B: Structure, sum formula and theoretical exact mass of Darobactin A with plausible MS/MS fragmentation pattern..16
Figure S3C: Structure, sum formula and theoretical exact mass of Darobactin B with plausible MS/MS fragmentation pattern..17
Figure S3D: Structure, sum formula and theoretical exact mass of Darobactin D with plausible MS/MS fragmentation pattern..18
Figure S3E: Structure, sum formula and theoretical exact mass of Darobactin E with plausible MS/MS fragmentation pattern..19
Table S1: ^{1}H (600 MHz) and ^{13}C (150 MHz) NMR data of Darobactin B (D_2O; δ in ppm) alongside correlation data from HMBC measurement 20
Figure S4: Structure of Darobactin B including atom numbering for table S1.......................22
Figure S5A: ^{1}H-NMR spectrum of Darobactin B (600 MHz, D_2O). 23
Figure S5B: 24
Expanded ^{1}H-NMR spectrum of Darobactin B (600 MHz, D_2O)..24
Figure S5C: ^{13}C-NMR spectrum of Darobactin B (150 MHz, D_2O) 25
Figure S5D: COSY spectrum of Darobactin B in D_2O...27
Figure S5E: TOCSY spectrum of Darobactin B in D_2O 28
Figure S5F: HSQC spectrum of Darobactin B in D_2O...29
Figure S5G: HMBC spectrum of Darobactin B in D_2O..30
Figure S5H: NOESY spectrum of Darobactin B in D_2O 31
Figure S5I: ROESY spectrum of Darobactin B in D_2O 32
Table S2: Oligonucleotide sequences used to modify the DAR A core AAs in pNBDaromod .33
Table S3A: Antibiogram of the clinical isolates 33
Table S3B: Resistance determinants of the clinical isolates 34
Table S4: Statistics on X-ray diffraction data and refinement of BamA-β/Darobactin B........34

Isolation of compounds

Purification of DAR B from the producer strain was achieved with a modified purification strategy from DAR A. Briefly, *E. coli* production strains were incubated for 5 days in a 2 L Erlenmeyer flask with 1 L LB medium supplemented with 50 µg/mL kanamycin at 30 °C. Cells were removed via centrifugation and the culture supernatant was mixed with XAD16N resin (Sigma-Aldrich) overnight under agitation. DAR B was subsequently eluted from the resin with a 50/50 solution of methanol and water, containing 0.1% formic acid. The eluate was then concentrated via rotary evaporator and loaded onto a cation-exchange column (SP Sepharose XL). DAR B was eluted by step gradients of 50 mM ammonium acetate pH 7, pH 8, and pH 10. Eluates were then concentrated by freeze drying, resuspended in Milli-Q water 0.1% (v/v) formic acid, and loaded onto a C_{18} reversed-phase high-performance liquid chromatography (RP-HPLC) column (Agilent, C18 5 µm: 250 x10mm, Restek). HPLC conditions for purification of DAR B are: solvent A, Milli-Q water and 0.1% (v/v) formic acid; solvent B, acetonitrile and 0.1% (v/v) formic acid. The initial concentration of 2% solvent B is maintained for 2 min, followed by a linear gradient to 26% B over 12 min with a flow rate of 5 mL min^{-1}; UV detection by diode-array detector from 210 to 400 nm. Pure DAR B was then collected at 11.5 min.

For purification of DAR E, fermentation broth was pelleted by centrifugation. The cell pellet was extracted using 80% acetonitrile and water by sonification. The resulting crude extract was fractionated by flash chromatography using a C18 F0120 column with the following gradient: 1) 0-28min 5%ACN, 2) 28-37min increased to 15% ACN, 3) 37-50 min, keeping 15% ACN, 4) 50-60min, increased to 30% ACN, 5) 60-80min, increased to 100% ACN and keeping 100% ACN for 15 min. By LCMS guided isolation, the DAR E-containing fraction was identified and further separated by HPLC using the following gradient: 1) 0-10 min 23% MeOH, 2)10-20min increased to 50% MeOH, 3) 20-30 min increased to 100% MeOH, 4) 30-37min 100%MeOH. Afterwards, the DAR E fraction was further purified by HPLC (gradient: 1) 0-5min 25%MeOH, 2) 5-45 min increased to 42.5% MeOH, 3) 45-52min keeping 100% MeOH to obtain pure compound (in total 1.3 mg DAR E was isolated from a 98 L fermentation.

For DAR D the same procedure via flash chromatography was followed. Then, the following HPLC gradient was applied: 1) 0-5 min 15% ACN, 2) 5-25 min increased to 25% ACN, 3) 25-30 min increased to 60% ACN, 4) 30-39 min 100% ACN. As before, a further HPLC separation followed to obtain DAR D as pure compound (in total, 0.6 mg DAR were isolated from a 97 L fermentation.

Structure elucidation

Production of DAR A, B, D, E and F was confirmed using ultra high-performance liquid-chromatography high-resolution mass-spectrometry (UPLC-HRMS). For UPLC-HRMS measurements a setup consisting of an Agilent Infinity 1290 UPLC system equipped with an Acquity UPLC BEH C18 1.7 µm (2.1 × 100 mm) column and an Acquity UPLC BEH C18 1.7 µm VanGuard Pre-Column (2.1 × 5 mm; both columns purchased from Waters, Eschborn, Germany) coupled to a DAD detector and a micrOTOFQ II mass spectrometer (Bruker Daltonics, Bremen, Germany) with an electrospray ionization source was employed. The LC system was operated using a gradient (A: H_2O, 0.1% formic acid; B: MeCN, 0.1% formic acid; Flow: 600 µL/min): 0 min: 95%A; 0.80 min: 95%A; 18.70 min: 4.75%A; 18.80 min: 0%A; 23.00 min: 0%A; 23.10 min: 95%A; 25.00 min: 95%A and the column oven temperature was set to 45 °C.

MS data were acquired over a range from 100 to 1500 *m/z* in positive mode. Auto MS/MS fragmentation was achieved with rising collision energy (for single charged ions: 18–45 eV over a gradient from 100 to 1000 m/z; for double charged ions: 15–32 eV over a gradient from 100 to 1000 m/z). Calibration of mass spectra was achieved using 10 mM sodium formate in H_2O/ ^{i}PrOH (1:1) as internal standard.

Mass spectra were analysed using Bruker Data Analysis 4.2 software (Bruker Daltonics, Bremen, Germany). Masses of the expected compounds were determined using ChemDraw (PerkinElmer, Waltham, USA) and recorded chromatograms were extracted for the respective *m/z*. Ions with fitting (Δ<10 ppm) *m/z* and plausible retention time were fragmented and fragments were matched to *in silico* identified plausible decay products (Fig. S9-S13).

NMR spectra were recorded in D_2O as solvent on an Avance III HD 600 MHz NMR spectrometer (Bruker BioSpin GmbH, Rheinstetten, Germany). Chemical shifts are given in ppm. ^{1}H spectra were referenced to the residual solvent signal (δ = 4.79 ppm). For ^{13}C measurements 3-(trimethylsilyl)propionic-2,2,3,3-d_4 acid sodium salt (TSPA, δ = 1.7 ppm) was used as external standard. For a better resolution of the correlation signals, HMBC spectra were measured using non-uniform sampling (NUS) and H_2O suppression. Analysis of NMR spectra was accomplished using the software TopSpin 3.6.0 (Bruker BioSpin GmbH, Rheinstetten, Germany).

Minimum inhibitory concentration testing

The MICs were determined by microbroth dilution assays in round bottom 96-well plates. Overnight cultures of *E. coli* ATCC35218 and the clinical isolates *E .coli* (Table S2) NRZ14408, *E. coli* K0416, *E. coli* Survcare 052, *E. coli* MMGI1; *A. baumanii*

ATCC19606, *K. pneumoniae* ATCC30104 and *S. enterica* ATCC13076 were adjusted to McFarland 1.0 and subsequently diluted to 5 x 10^5 c.f.u. mL^{-1} in cation adjusted Mueller Hinton 2 broth (CAMHII). Darobactins were screened in 12 concentrations ranging from 64 to 0.03 µg mL^{-1} in triplicate. The same concentrations were tested for rifampicin, tetracycline and gentamycin as positive controls. For tetracycline resistant *E. coli* strains (NRZ14408, K0416 and MMGI1) as well as for *E. coli* Survcare 052 tetracycline was substituted with a colistin dilution series (16 – 0.007 µg mL^{-1}).

Bacteria suspension without supplemented standard antibiotics or Darobactin was used as negative control. After incubation (18 h, 180 rpm, 37°C, 85 % r.H.), cell growth was determined by measuring the turbidity with a microplate spectrophotometer at 600 nm. The MIC was defined as the minimum concentration where at least 85 % growth inhibition relative to the negative control was measured.

MICs against the expanded panel (Table 3) were determined by microbroth dilution as well, but without shaking. In aerobic conditions, overnight cultures of *K. pneumoniae* strains, *E. coli* strains, *P. aeruginosa* strains, *A. baumanii* strains, *M. catarrhalis*, *E. cloacae, P. mirabilis, S. maltophilia, S. aureus* were diluted 1:100 in MHB and incubated at 37 °C with aeration at 220 rpm. Exponential cultures (OD_{600} of 0.1–0.9) were diluted to an OD_{600} of 0.001 (approximately 5 × 10^5 c.f.u. mL^{-1}) in MHIIB and 98 µL aliquots were transferred into round-bottom 96-well plates containing 2 µL of test compound solutions diluted serially two-fold. After overnight incubation at 37 °C, the MICs of test compounds were determined as the minimum concentration at which no growth of strains could be detected by eye. The MIC against *Salmonella enterica* Typhimurium LT2 and *Bacteroides fragilis* was determined under anaerobic conditions. Overnight cultures grown in brain–heart infusion (BHI) broth, supplemented with 0.5% yeast extract, 0.1% L-cysteine hydrochloride and 15 µg/mL haemin (BHI-Ych) were diluted 1:100 in BHI-Ych. The 96-well assay plates were prepared by two-fold dilution of test compounds and included a positive growth control. After 24 h incubation, the MIC was determined. All MIC assays were performed at least in triplicate.

Checkerboard Assays

Checkerboard assays were performed by determining the MIC in combination of DAR A and DAR B by microbroth dilution assays in 96-well plates. Concentrations ranging from 0 to 64 µg/mL of both compounds were tested in duplicate. Overnight

cultures of *E. coli* MG1655 and *K. pneumoniae* ATCC 700603 were diluted to 5 x 10^5 c.f.u. mL^{-1} in MHIIB. After overnight incubation at 37 °C, the MIC of test compounds were determined as the minimum concentration at which no growth of strains could be detected by eye. Fractional inhibitory concentration index (FICI) was calculated by FICA + FICB = FICI, where FICA = MIC DAR A+B ÷ MIC DAR A, and FICB = MIC DAR A+B ÷ MIC DAR B. Synergy, additive, and antagonistic effects were determined by FICI values of <0.4, 0.4-4, and >4, respectively.

Plasma Protein Binding

Plasma protein binding was determined by a Rapid Equilibrium Dialysis (RED) plate (Fisher 90006). The assay was performed following Bioassay Protocol 1937 (1). Briefly, DAR A and DAR B were mixed with human plasma (300 µL) at a final concentration of 10 µM. 500 µL PBS was added to the buffer chamber. The RED plate was sealed, and incubated at 37 °C at 300 rpm for 4 hours. 100 µL were removed from the sample and buffer chambers. Then, 100 µL plasma was added to the buffer samples, and 100 µL PBS was added to the plasma samples. 400 µL of acetonitrile was added, vortexed, and centrifuged for 10 minutes at 13,000 g. The samples were dried, and then resolubilized in 100 µL MilliQ water prior to quantitative measurements on LC-MS/MS. An Agilent 1260 Infinity liquid chromatography system coupled to a 6530 quadrupole time-of-flight (QTof) mass spectrometer equipped with an electrospray ionization source (Agilent Technologies) was used to quantify DAR A and DAR B. An Agilent ZORBAX Extend-C18 column (50 mm × 2.1 mm, 1.8 µm) was utilized for the separation with a flow rate of 100 µL/min with solvent A (0.1% (v/v) formic acid in Milli-Q water) and solvent B (0.1% (v/v) formic acid in acetonitrile). The initial concentration of 2% solvent B was maintained for 2 min, followed by a linear gradient to 95% over 7 min. MS parameters were as follows: gas temperature, 300 °C; gas flow, 7 L/min; nebulizer 35 psi; fragmentor voltage, 175 V; skimmer voltage, 65 V. Acquisition was set in positive mode at “Targeted MS/MS” with a precursor m/z 483.7, z = 2, collision energy 12 V for DAR A and a precursor m/z 350.15, z = 3, collision energy 11 V for DAR B. MassHunter qualitative analysis (Agilent Technologies) and Skyline (MacCoss Lab Software, University of Washington) were used to perform relative quantification of DAR A and DAR B (2).

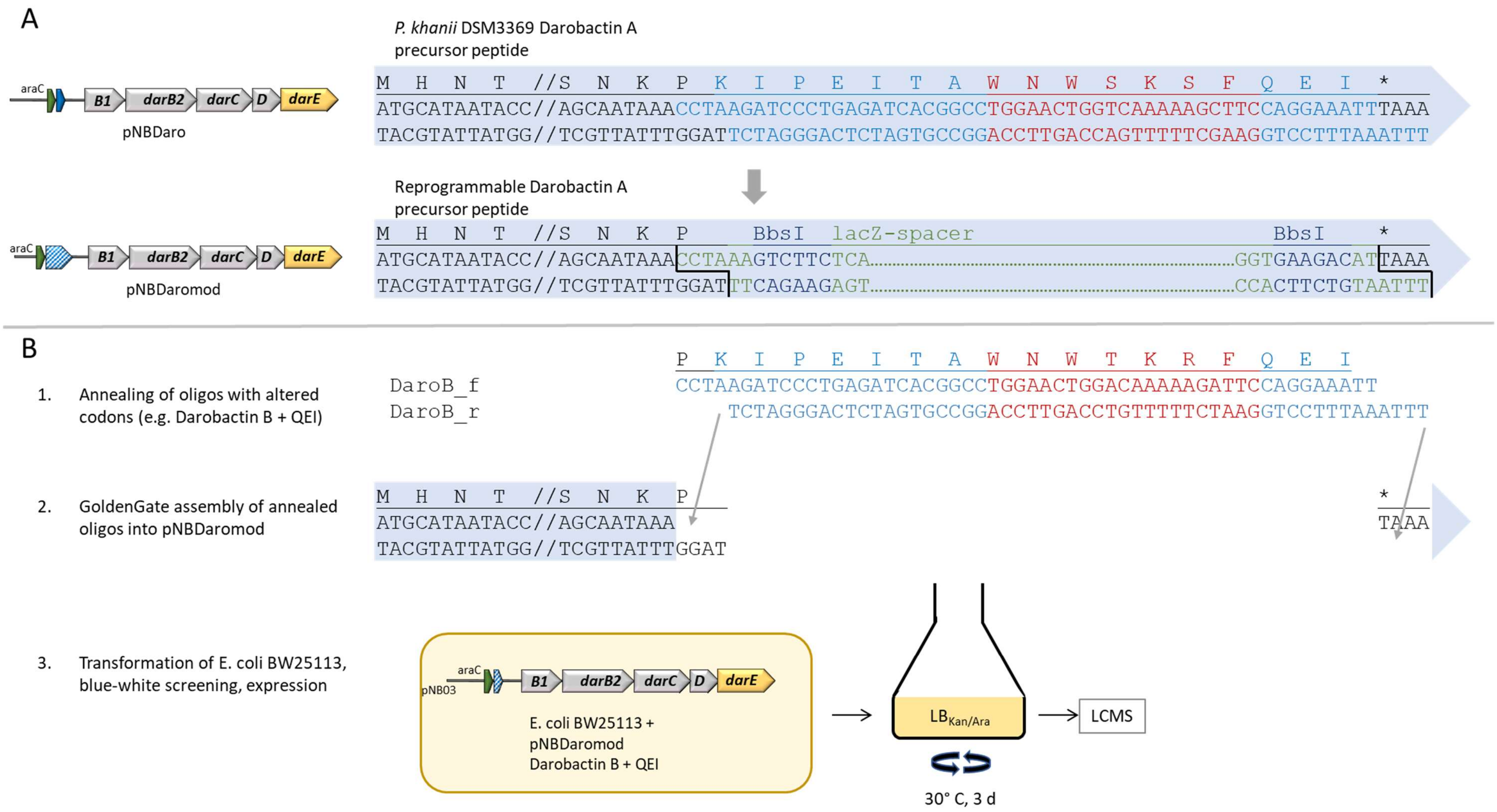

Figure S1: General cloning strategy for exchange of core AAs in the DAR A precursor. A) replacement of the core AAs with a pCRISPOMYCES2 derived lacZ spacer. B) individual replacement of core AAs in the DAR A precursor by GoldenGate assembly using annealed synthesized oligonucleotides and subsequent cultivation of strains carrying modified plasmids.

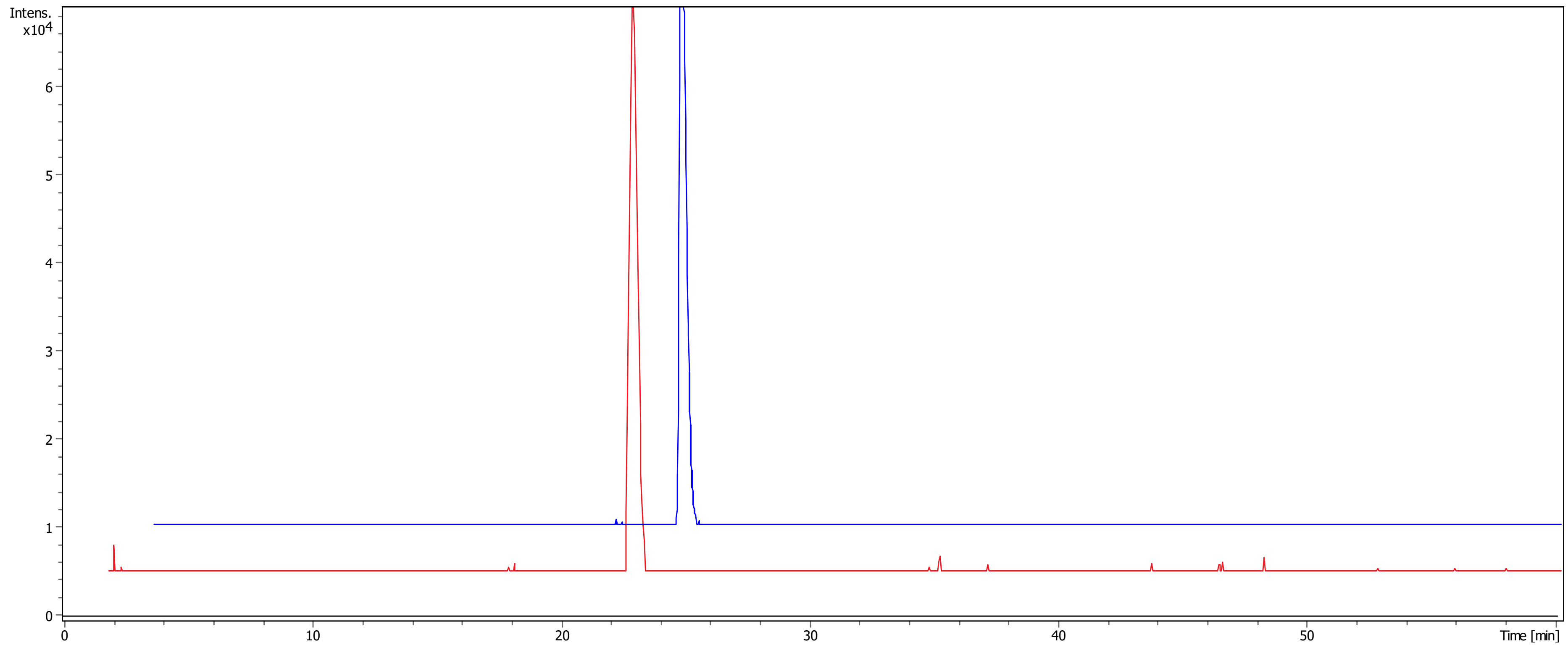

Figure S2A: Extracted ion chromatogram (EICs, calcd. $[M + 2H]^{2+}$ ± 0.01 Da) of DAR A (483.7089 *m/z*) for *E. coli* BW25113 + pNBDarC (- crtl, black) *E. coli* BW25113 + pNB03-darA-E (incl. follower, red, Imai *et al.,* 2019) and *E. coli* BW25113 + pNBDarA (-follower, blue).

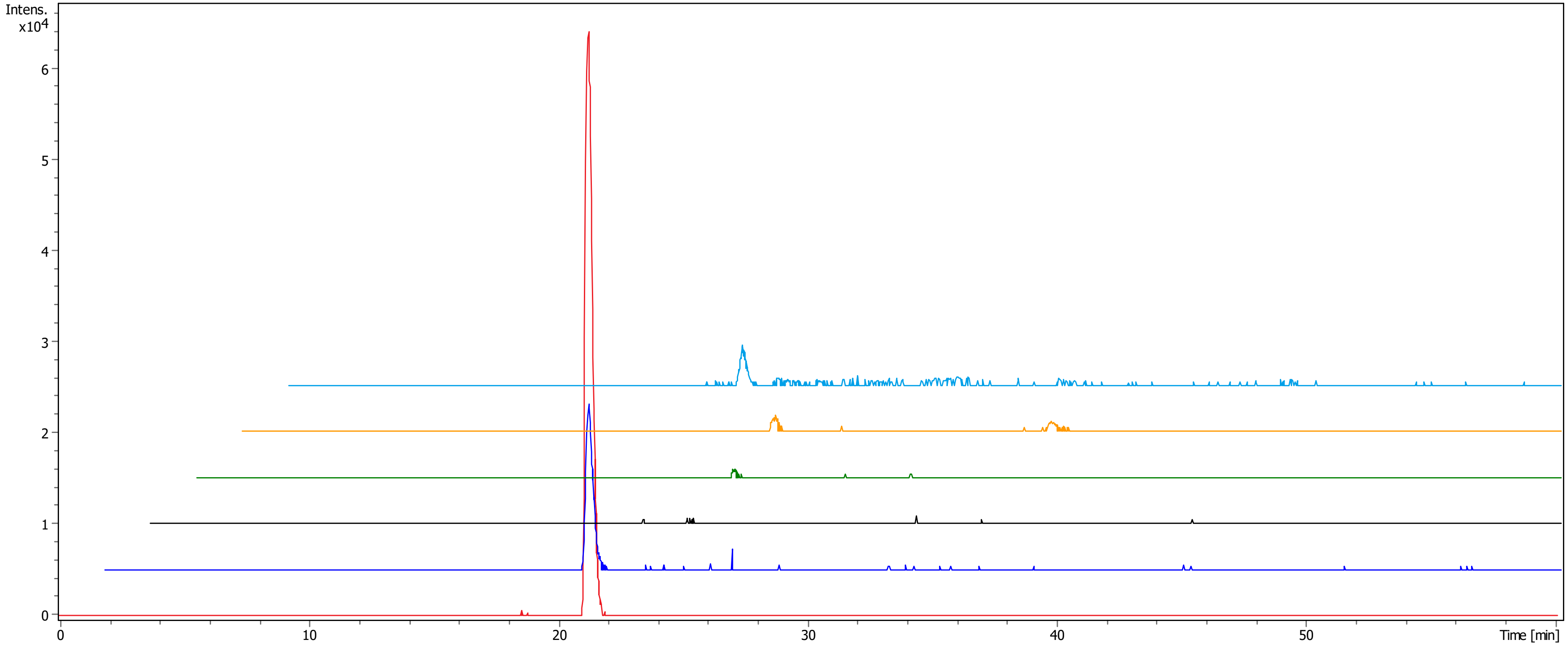

Figure S2B: Extracted ion chromatograms (EICs, calcd. $[M + 2H]^{2+} \pm 0.01$ Da) of DAR A (483.7089 m/z) and the DAR analogues B (525.2512 *m/z*), C (484.2065 *m/z*), D (497.7119 *m/z*), E (470.2034 *m/z*) and F (487.2482 *m/z*) from bottom to top.

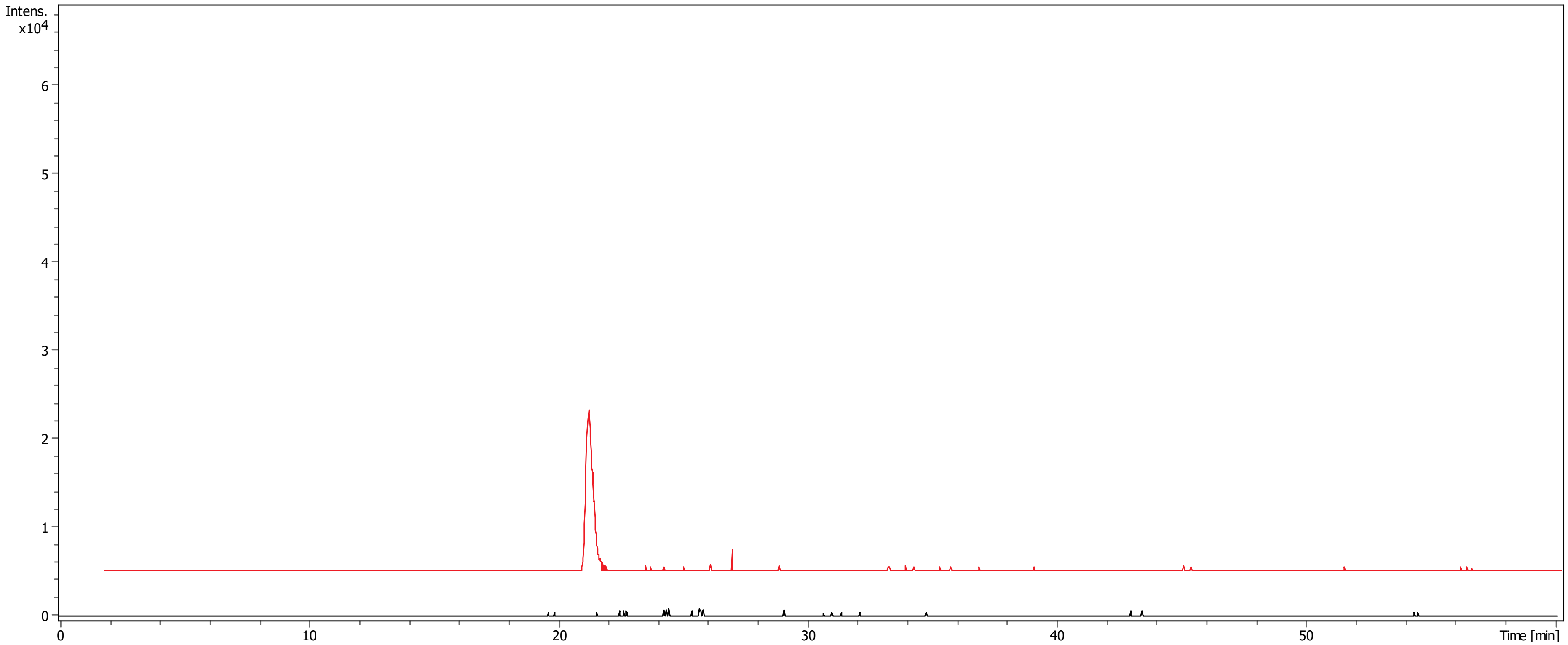

Figure S2C: Extracted ion chromatogram (EICs, calcd. $[M + 2H]^{2+}$ ± 0.01 Da) of DAR B (525.2512 *m/z*) for *E. coli* BW25113 + pNBDarA (- crtl, black) and *E. coli* BW25113 + pNBDarB (red).

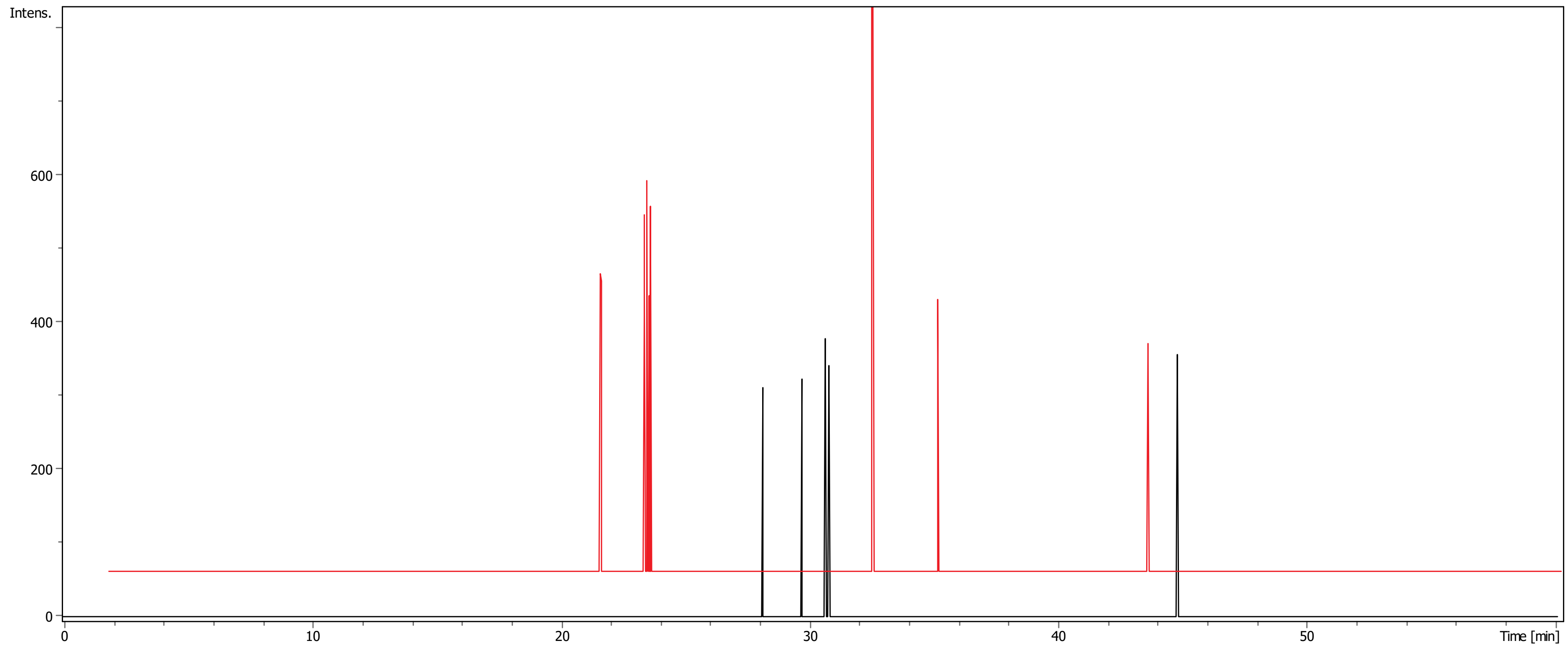

Figure S2D: Extracted ion chromatogram (EICs, calcd. $[M + 2H]^{2+}$ ± 0.01 Da) of DAR C (484.2065 *m/z*) for *E. coli* BW25113 + pNBDarD (- crtl, black) and *E. coli* BW25113 + pNBDarC (red).

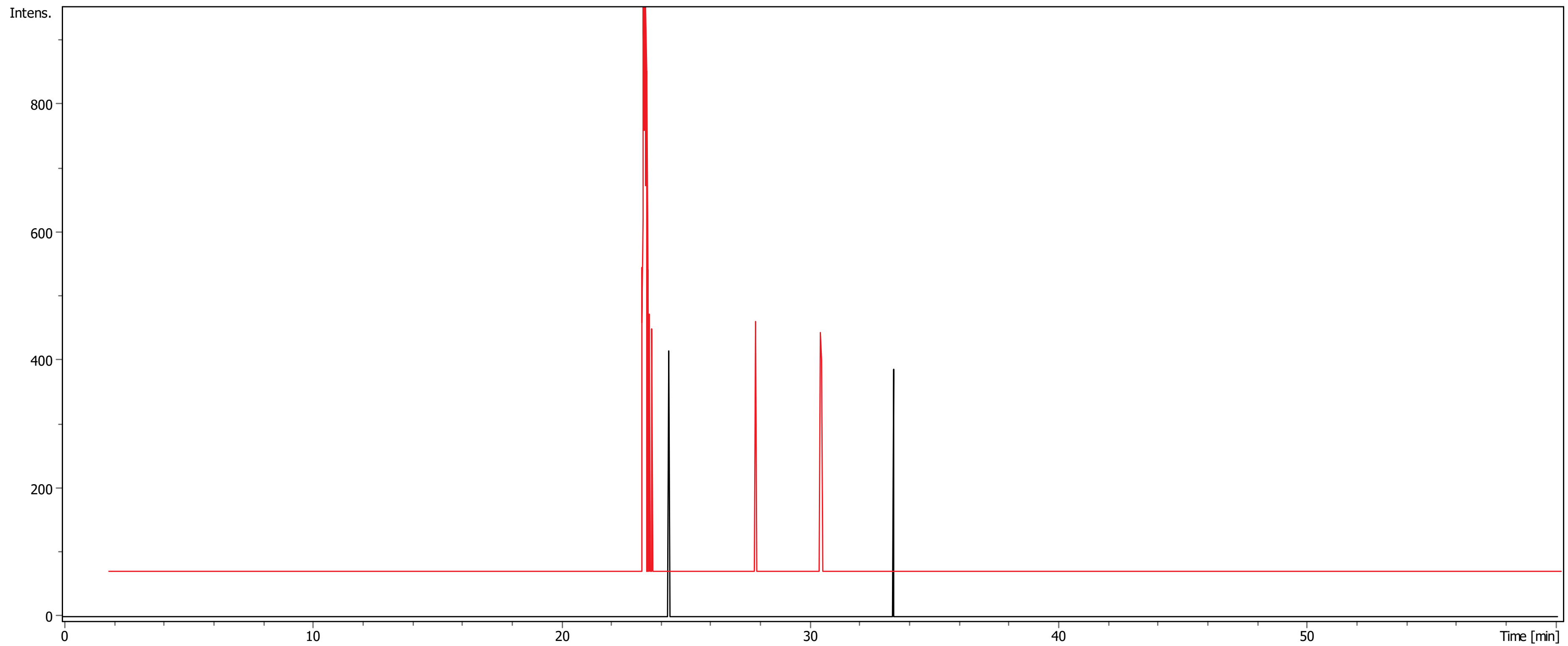

Figure S2E: Extracted ion chromatogram (EICs, calcd. $[M + 2H]^{2+}$ ± 0.01 Da) of DAR D (497.7119 *m/z*) for *E. coli* BW25113 + pNBDarA (- crtl, black) and *E. coli* BW25113 + pNBDarD (red).

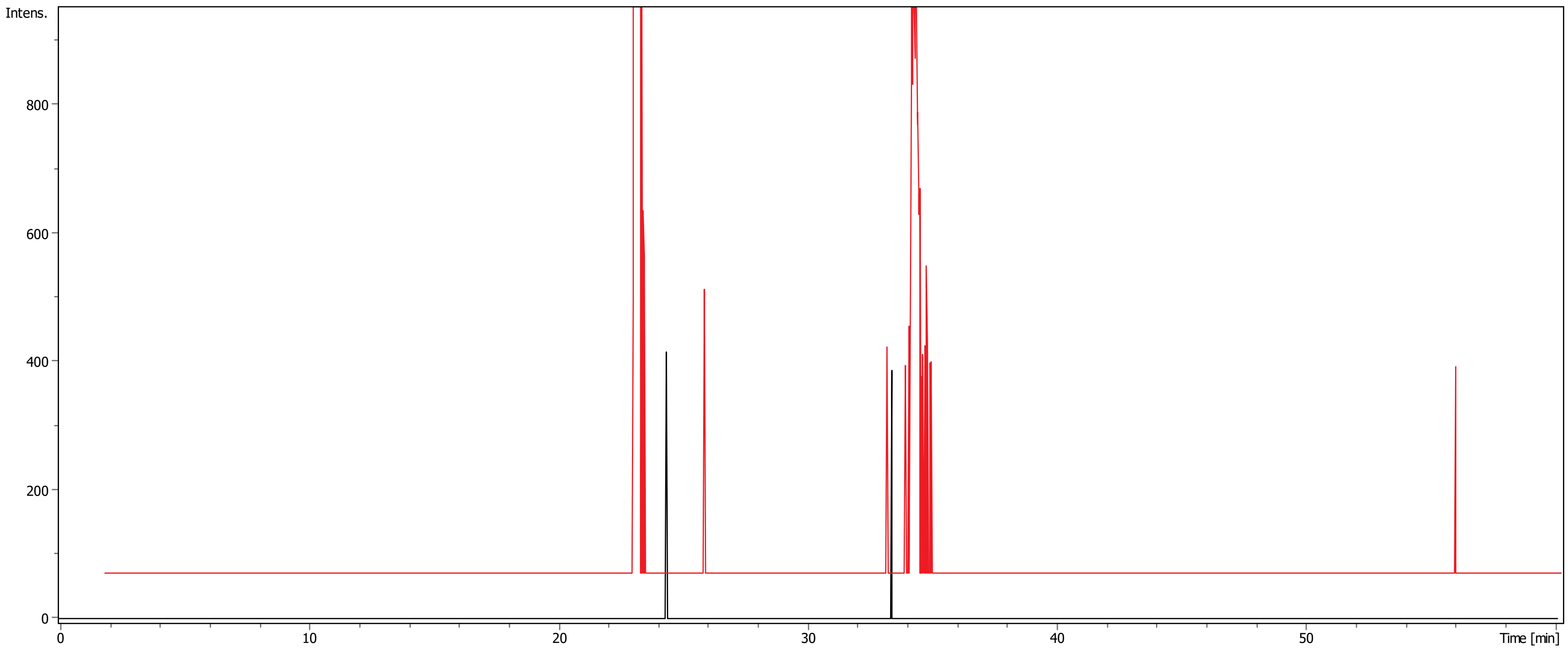

Figure S2F: Extracted ion chromatogram (EICs, calcd. $[M + 2H]^{2+}$ ± 0.01 Da) of DAR E (470.2034 *m/z*) for *E. coli* BW25113 + pNBDarA (- crtl, black) and *E. coli* BW25113 + pNBDarE (red).

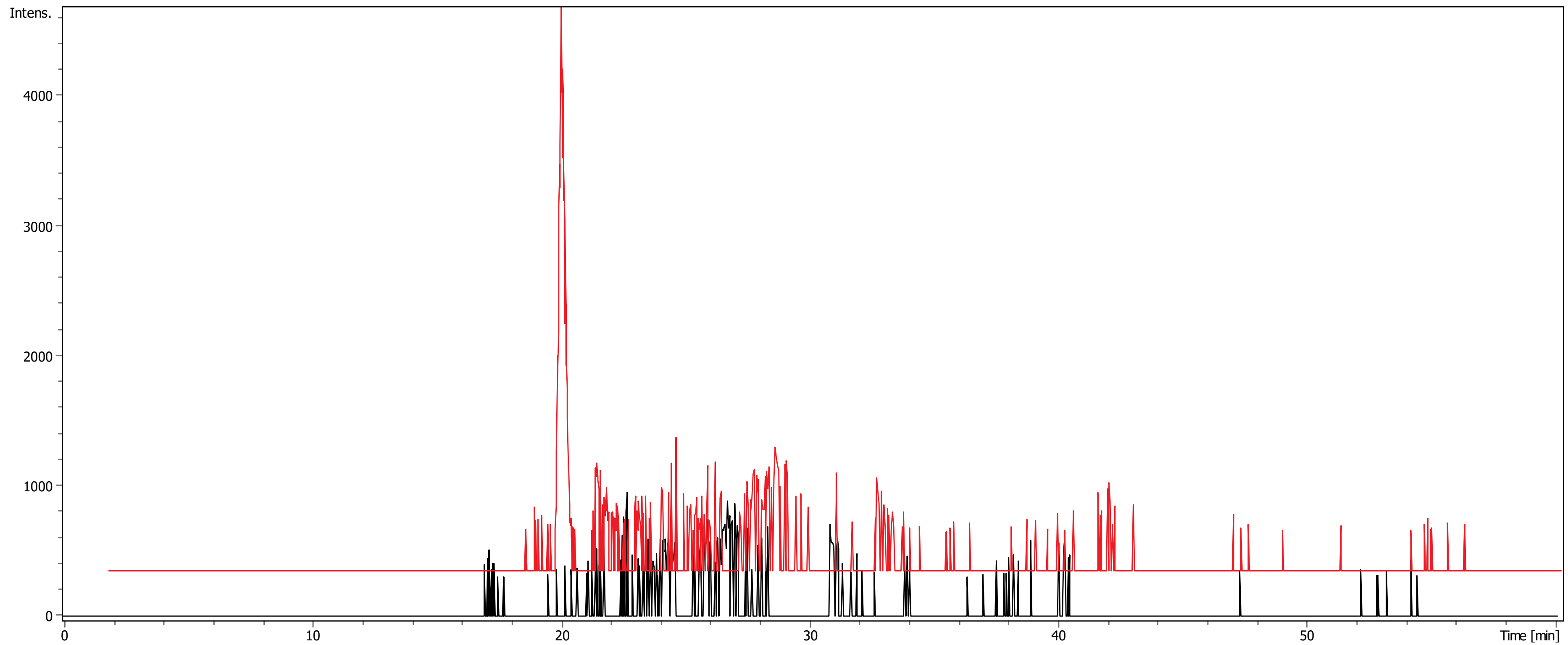

Figure S2G: Extracted ion chromatogram (EICs, calcd. $[M + 2H]^{2+}$ ± 0.01 Da) of DAR F (487.2482 *m/z*) for *E. coli* BW25113 + pNBDarA (- crtl, black) and *E. coli* BW25113 + pNBDarF (red).

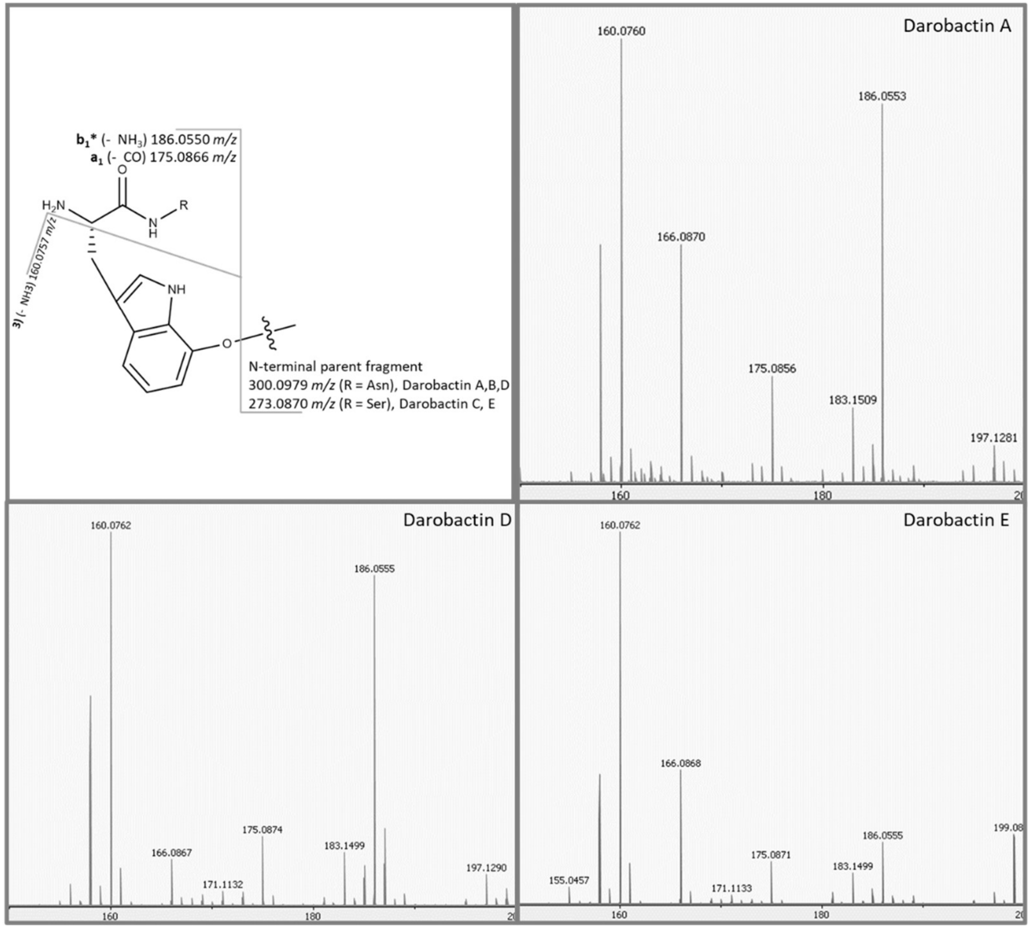

Figure S3A: Structure of the N-terminal W of Darobactin Analogues with plausible MS/MS fragmentation pattern and MS/MS spectra of Darobactin A, D and E. Respective measured fragment ions are annotated. Darobactin B was measured on a different instrument that did not measure < 200 *m/z*.

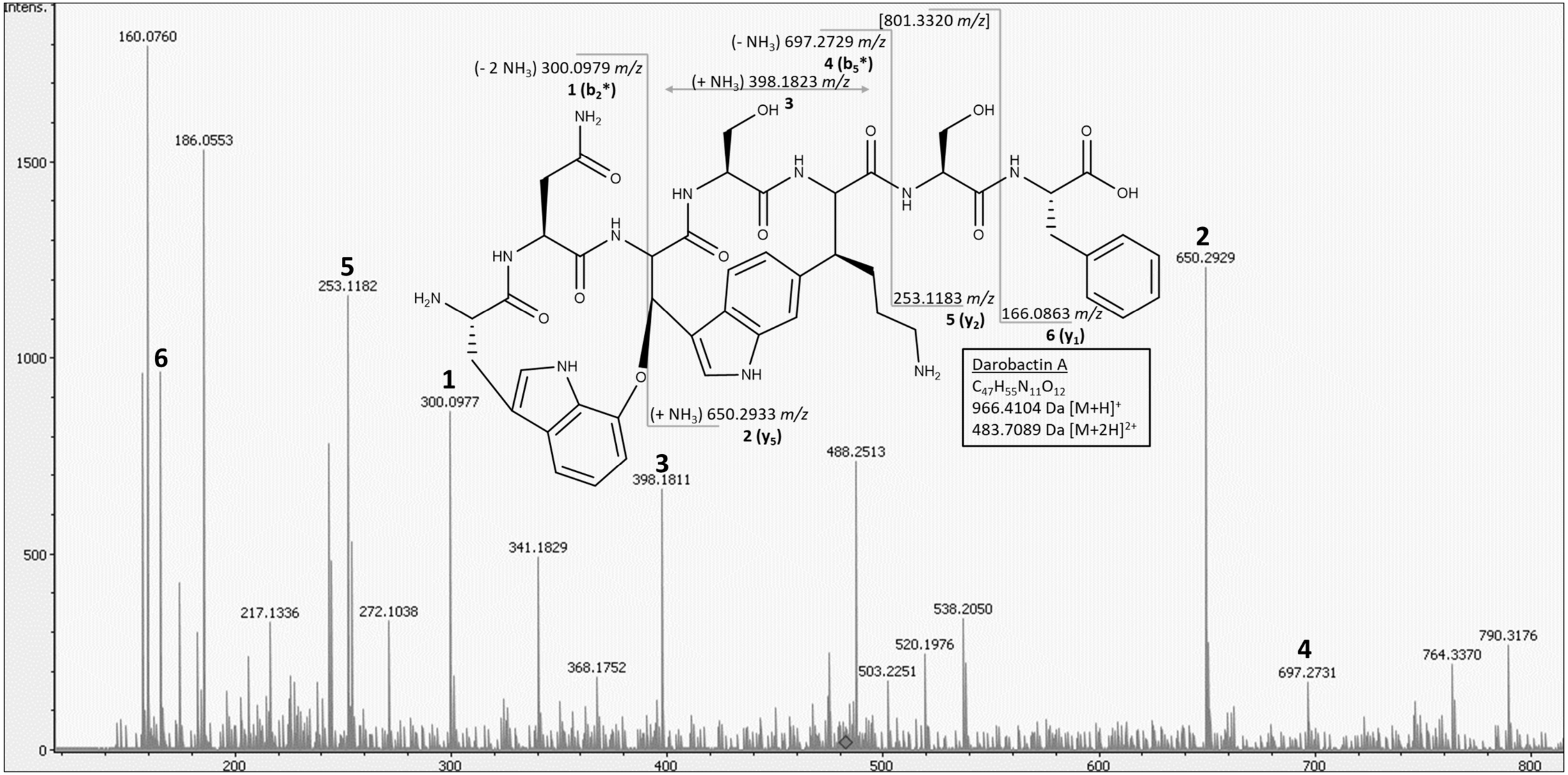

Figure S3B: Structure, sum formula and theoretical exact mass of Darobactin A with plausible MS/MS fragmentation pattern and MS/MS spectrum. The respective measured fragment ions that correspond to the shown fragmentation pattern are annotated.

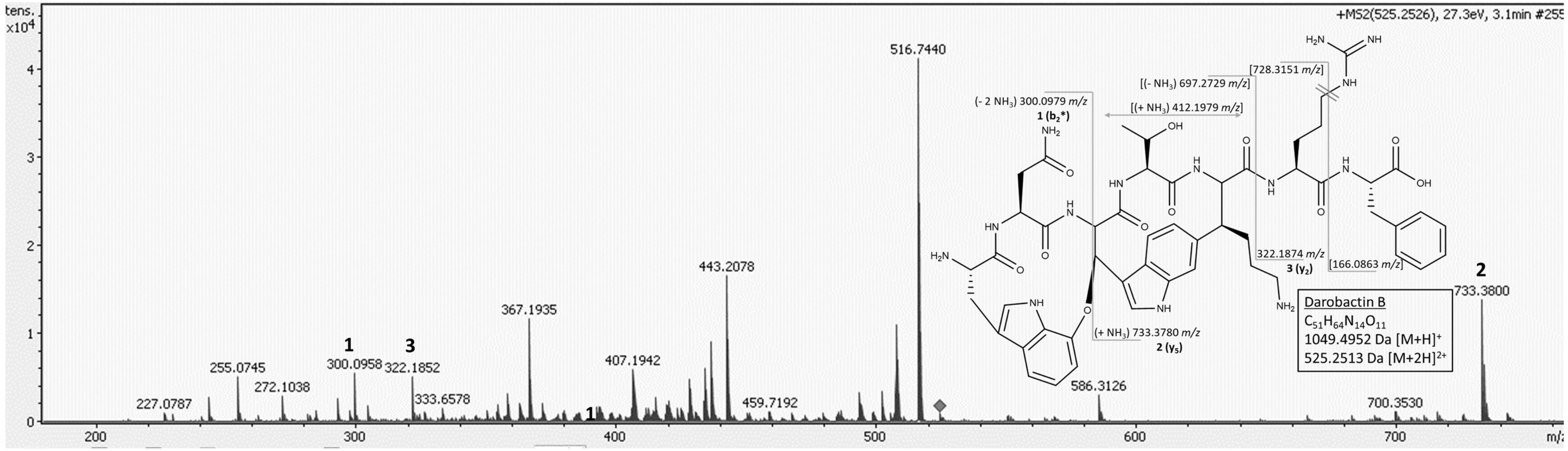

Figure S3C: Structure, sum formula and theoretical exact mass of Darobactin B with plausible MS/MS fragmentation pattern and MS/MS spectrum. The respective measured fragment ions that correspond to the shown fragmentation pattern are annotated.

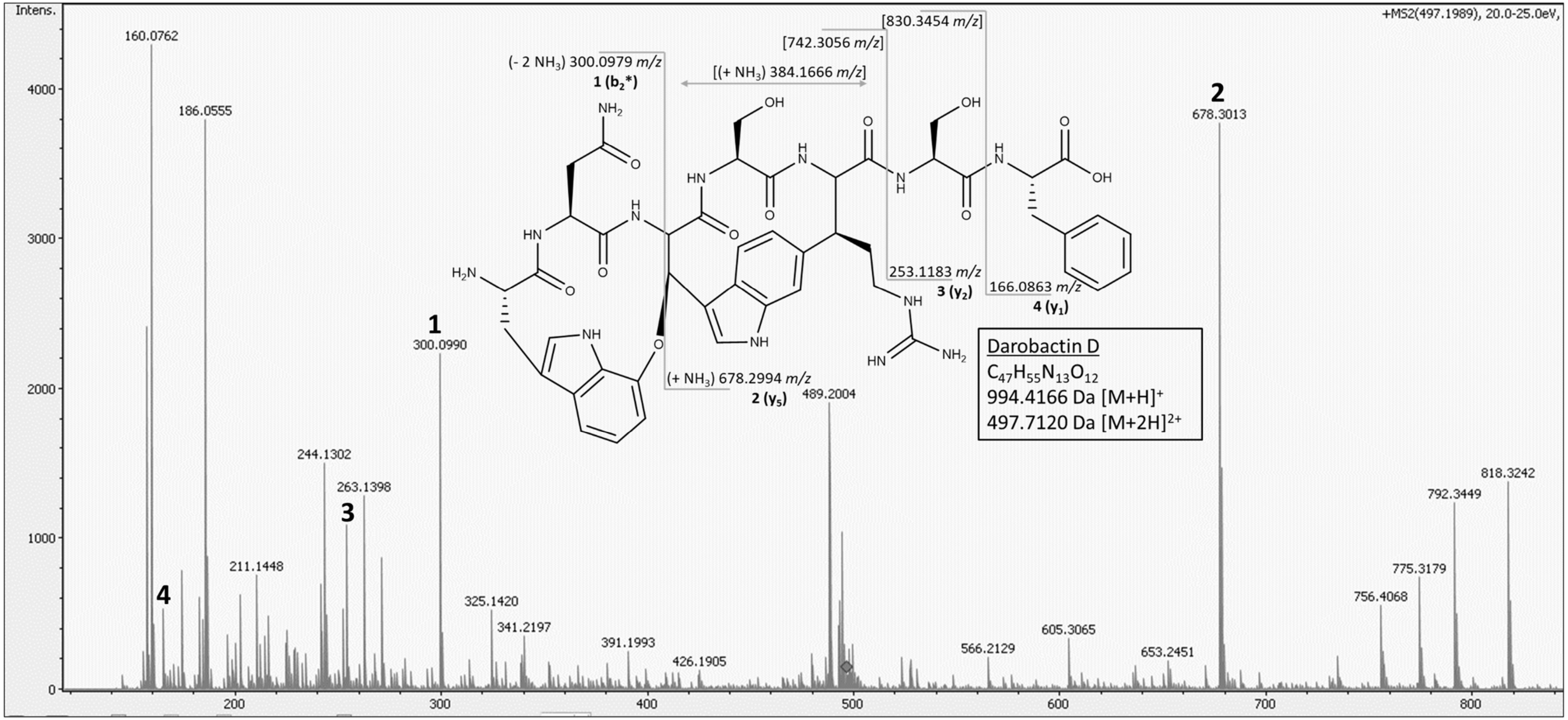

Figure S3D: Structure, sum formula and theoretical exact mass of Darobactin D with plausible MS/MS fragmentation pattern and MS/MS spectrum. The respective measured fragment ions that correspond to the shown fragmentation pattern are annotated.

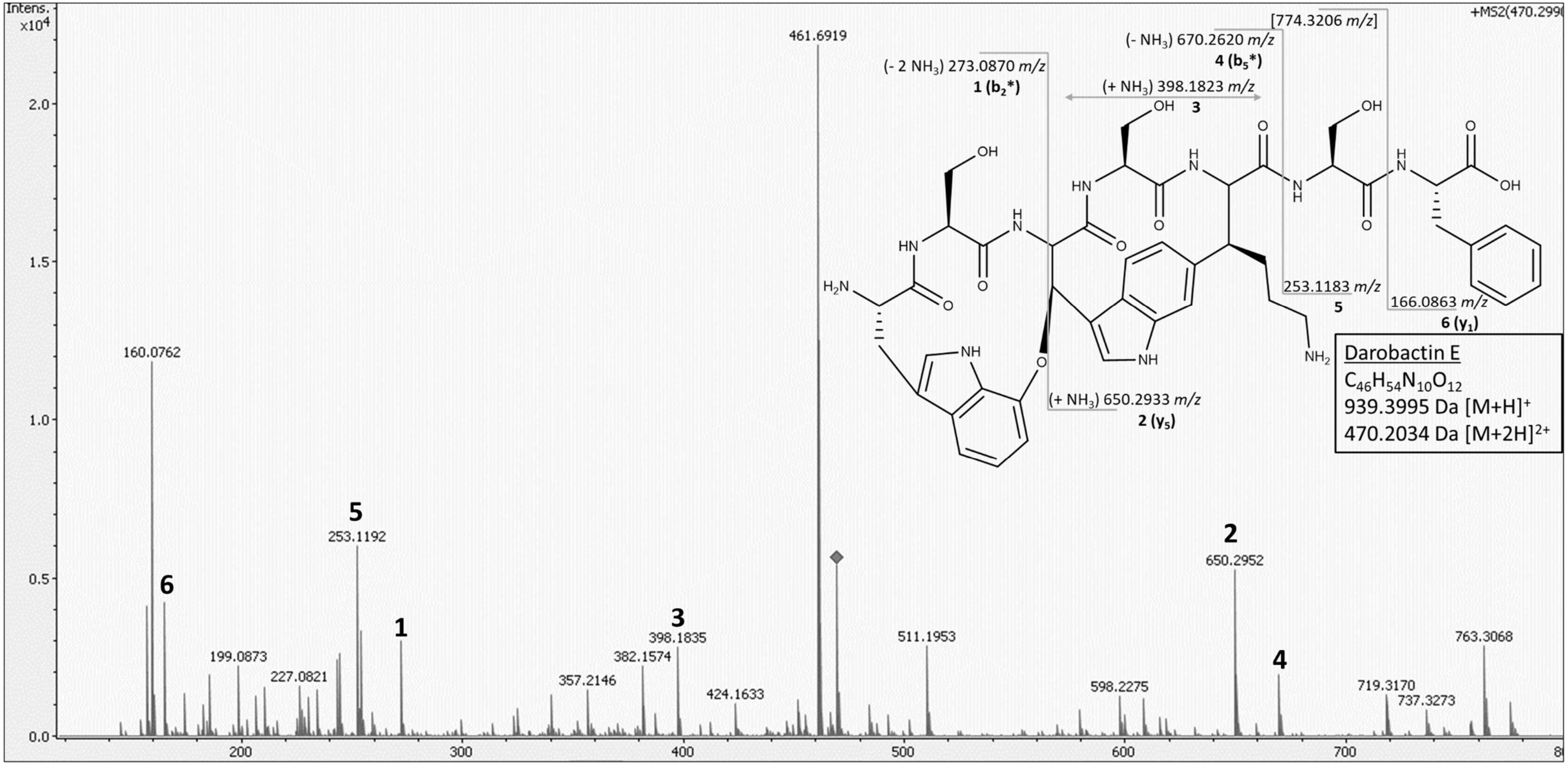

Figure S3E: Structure, sum formula and theoretical exact mass of Darobactin E with plausible MS/MS fragmentation pattern and MS/MS spectrum. The respective measured fragment ions that correspond to the shown fragmentation pattern are annotated.

Table S1: ^{1}H (600 MHz) and ^{13}C (150 MHz) NMR data of Darobactin B (D_2O; δ in ppm) alongside correlation data from HMBC measurement. For ^{13}C measurements 3-(trimethylsilyl)propionic-2,2,3,3-d_4 acid sodium salt (TSPA) was used as external standard. For atom numbering cf. Figure S4.

Amino acid	Position	δ_C [ppm], Type	δ_H [ppm], Mult. (J in Hz), ∫	HMBC coupling with (δ_C in ppm, corresponding amino acid)
Trp (N-terminal)	1	59.11, CH	4.08, dd (7.29, 10.95), 1H	2 (30.72, Trp), 11 (172.64, Trp)
	2	30.72, CH_2	3.60, dd (7.20, 13.68), 1H; 3.35, d (12.94), 1H	Trp 1 (59.11), Trp 3 (112.56), Trp 10 (133.46), Trp 11 (172.64)
	3	112.56, C_q	-	
	4	129.15, CH	7.43 – 7.36, m, 1H[a]	3 (112.56, Trp), 5 (133.50, Trp) or / and 10 (133.46, Trp)
	5	133.50, C_q	-	
	6	149.59, C_q	-	
	7 + 9	118.24, CH; 113.45, CH	7.30, pseudo-t (7.29), 2H	5 (133.50, Trp), 6 (149.59, Trp), 7 + 9 (118.24, 113.45, both Trp)
	8	124.68, CH	7.24, t (7.74), 1H	6 (149.59, Trp), 10 (133.46, Trp)
	10	133.46, C_q	-	
	11	172.64, C_q	-	
Asn	12	55.19, CH	3.37, t (6.84), 2H (expected: 1H)	11 (172.64, Trp), 13 (43.29, Asn), 14 (178.12, Asn), 15 (172.97, Asn),
	13	43.29, CH_2	2.24 - 2.17, m, 2H	12 (55.19, Asn), 14 (178.12, Asn), 15 (172.97, Asn)
	14	178.12, C_q	-	
	15	172.97, C_q	-	
Trp	16	67.81, CH	4.74, d (9.00), 1H	15 (172.97, Asn), 17 (81.34, Trp), 26 (172.59, Trp)
	17	81.34, CH	6.24, d (8.94), 1H	6 (149.59, Trp), 18 (116.09, Trp), 19 (128.88, Trp)
	18	116.09, C_q	-	
	19	128.88, CH	7.90, s, 1H	18 (116.09, Trp), 20 (141.58, Trp), 25 (129.28, Trp)
	20	141.58, C_q	-	
	21	114.95, CH	7.51, s, 1H	25 (129.28, Trp), 32 (52.40, Lys)
	22	137.41, C_q	-	
	23	129.34, CH	6.99, dd (0.99, 8.37), 1H	21 (114.95, Trp), 25 (129.28, Trp), 32 (52.40, Lys)
	24	121.87, CH	7.50 – 7.45, m, 1H[b]	18 (116.09, Trp), 20 (141.58, Trp), 22 (137.41, Trp)
	25	129.28, C_q	-	
	26	172.59, C_q	-	

Table S1 (continued)

Amino acid	Position	δ_C [ppm], Type	δ_H [ppm], Mult. (J in Hz), ∫	HMBC coupling with (δ_C in ppm, corresponding amino acid)
Thr	27	62.52, CH	3.77, d (6.48), 1H	26 (172.59, Trp) or/ and 30 (172.40, Thr), 28 (72.46, Thr), 29 (22.73, Thr)
	28	72.46, CH	3.43, p (6.38), 1H	-
	29	22.73, CH_3	0.83, d (6.42), 3H	27 (62.52, Thr), 28 (72.46, Thr)
	30	172.40, C_q	-	
Lys	31	64.51, CH	4.22, d (10.56), 1H	30 (172.40, Thr), 32 (52.40, Lys), 33 (30.13, Lys), 36 (175.85, Lys)
	32	52.40, CH	3.10 – 3.00, m, 1H[c]	-
	33	30.13, CH_2	2.16 – 2.09, m, 1H; 1.72 – 1.64, m, 1H[d]	-
	34	30.01, CH_2	1.98 – 1.89, m, 1H; 1.85 – 1.77, m, 1H	-
	35	43.84, CH_2	3.10 – 3.00, m, 2H[c]	-
	36	175.85, C_q	-	
Arg	37	57.81, CH	4.34, dd (6.12, 8.52), 1H	36 (175.85, Lys), 38 (32.73, Arg), 39 (28.83, Arg), 42 (177.43, Arg)
	38	32.73, CH_2	1.77 – 1.72, m, 1H; 1.72 – 1.64, m, 1H[d]	37 (57.81, Arg), 40 (44.88, weak, Arg)
	39	28.83, CH_2	1.58 – 1.45, m, 3H (expected: 2H)	37 (57.81, weak, Arg), 40 (44.88, Arg)
	40	44.88, CH_2	3.14, t (7.20), 2H	38 (32.73, Arg), 39 (28.83, Arg), 41 (161.13, Arg)
	41	161.13, C_q	-	
	42	177.43, C_q	-	
Phe	43	58.52, CH	4.77, dd (5.67, 9.03), 2H (expected: 1H)	42 (177.43, Arg), 44 (40.99, Phe), 45 (141.07, Phe), 49 (178.98, Phe)
	44	40.99, CH_2	3.33, dd (5.16, 13.90), 1H; 3.17, dd (8.88), 1H (overlay)	43 (58.52, Phe), 45 (141.07, Phe), 46, 46’ (133.86, Phe), 49 (178.98, Phe)
	45	141.07, C_q	-	
	46, 46’	133.86, CH	7.43 – 7.36, m, 2H[a]	44 (40.99, Phe), 46, 46’ (133.86, Phe), 48 (131.65, Phe)
	47, 47’	133.23, CH	7.50 – 7.45, m, 2H[b]	45 (141.07, Phe), 47, 47’ (133.23, Phe)
	48	131.65, CH	7.43 – 7.36, m, 1H[a]	46, 46’ (133.86, Phe)
	49	178.98, C_q	-	

[a] The observed integral for this signal was 4H due to the overlay of the proton signals for 4 (Trp), 46 (Phe), 46’ (Phe), and 48 (Phe). Thus, for each of these positions an integral of 1H was assigned. [b] The observed integral for this signal was 3H due to the overlay of the proton signals for 24 (Trp), 47 (Phe), and 47’ (Phe). Thus, for each of these positions an integral of 1H was assigned. [c] The observed integral for this signal was 4H due to the overlay of the proton signals for 32 (Lys), 35 (Lys) and traces of an impurity. The expected integral for the signal would be 3H, with 1H being ascribed to 32 (Lys) and 2H being assigned to 35 (Lys). [d] The observed integral for this signal was 3H due to the overlay of the proton signals for 33 (Lys), 38 (Arg) and traces of an impurity. The expected integral for the signal would be 2H, with 1H being ascribed to 33 (Lys) and 1H being assigned to 38 (Arg).

Figure S4: Structure of Darobactin B including atom numbering for table S1.

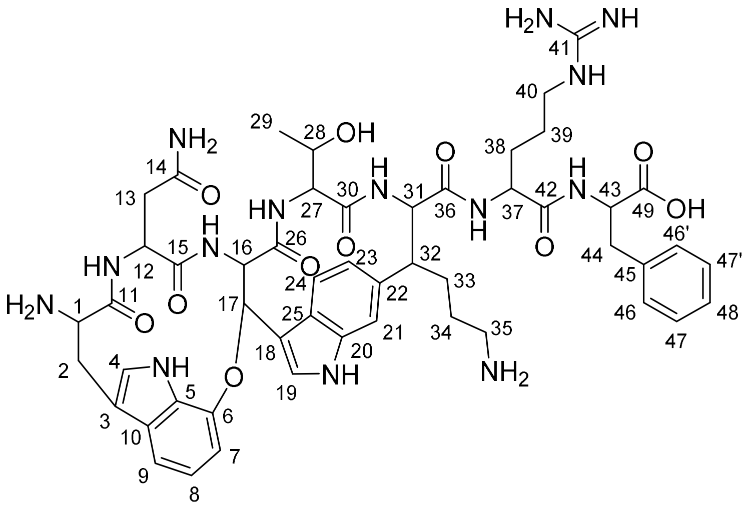

Figure S5A: ^{1}H-NMR spectrum of Darobactin B (600 MHz, D_2O).

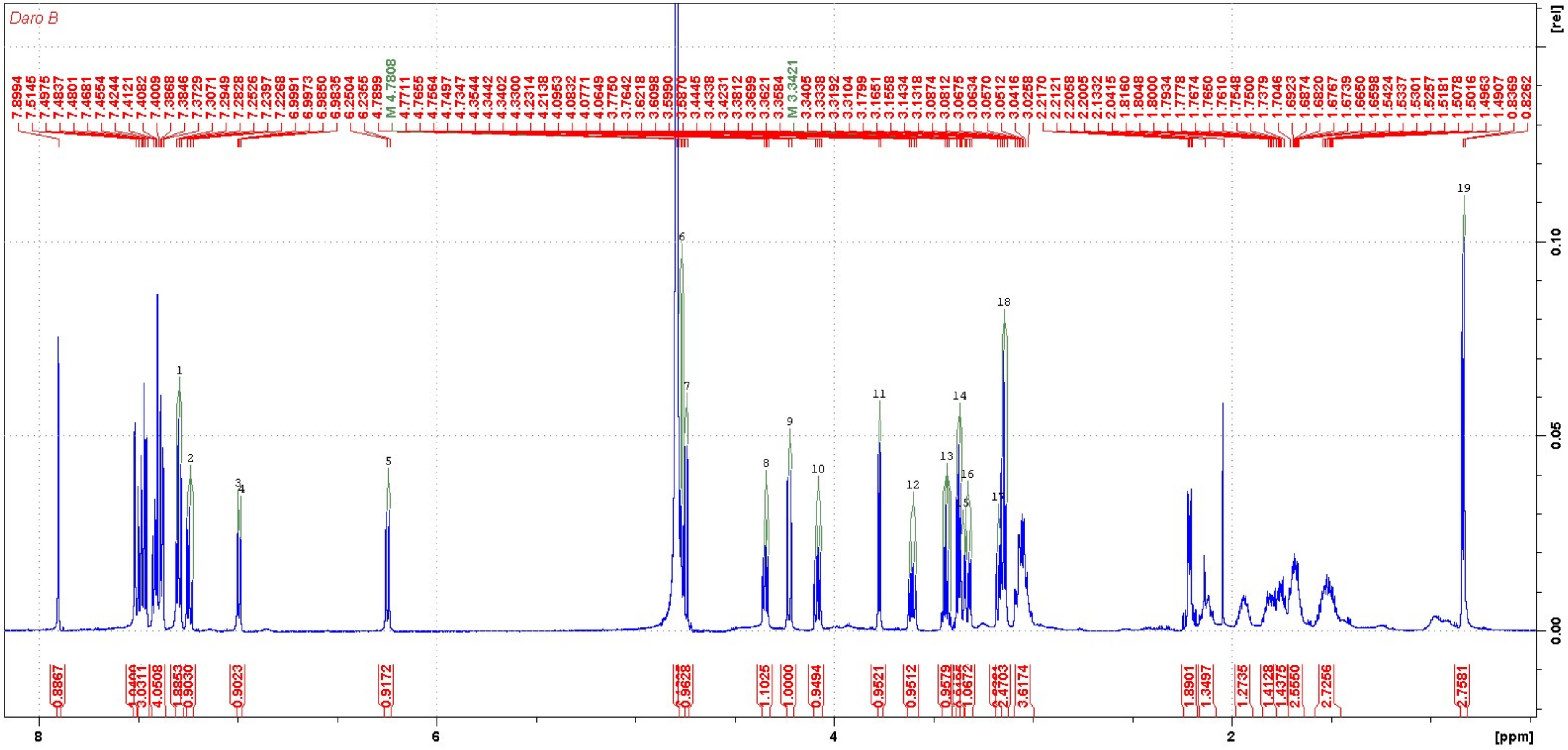

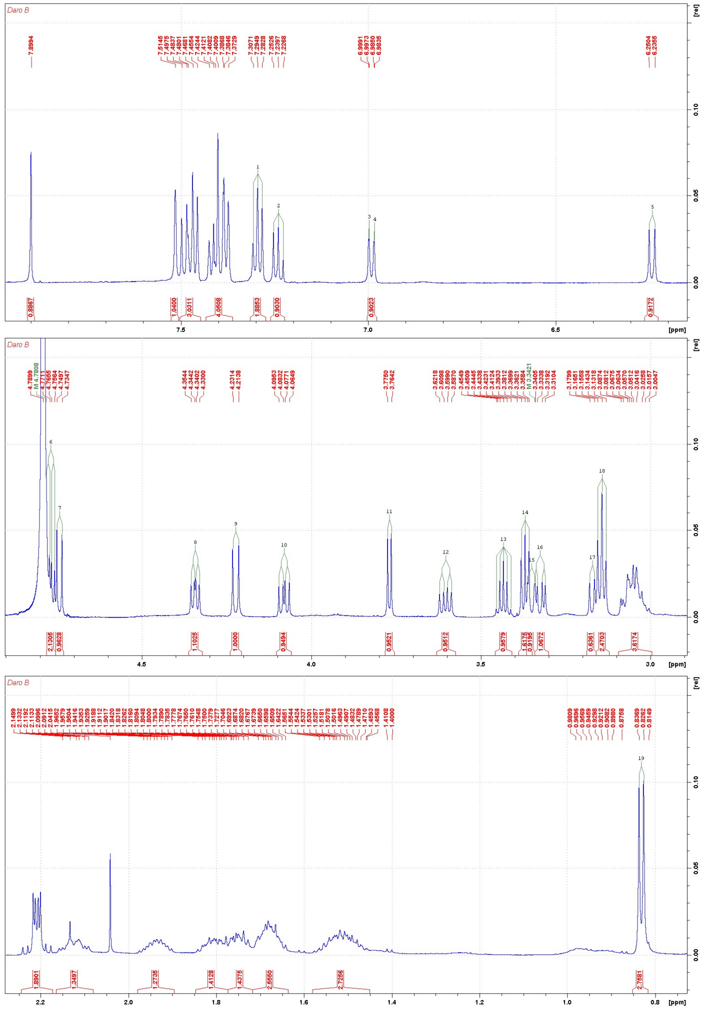

Figure S5B:

Expanded ^{1}H-NMR spectrum of Darobactin B (600 MHz, D_2O).

Figure S5C: ^{13}C-NMR spectrum of Darobactin B (150 MHz, D_2O). For the measurement 3-(trimethylsilyl)propionic-2,2,3,3-d_4 acid sodium salt (TSPA) was used as external standard. The multipletts indicate residual TFA.

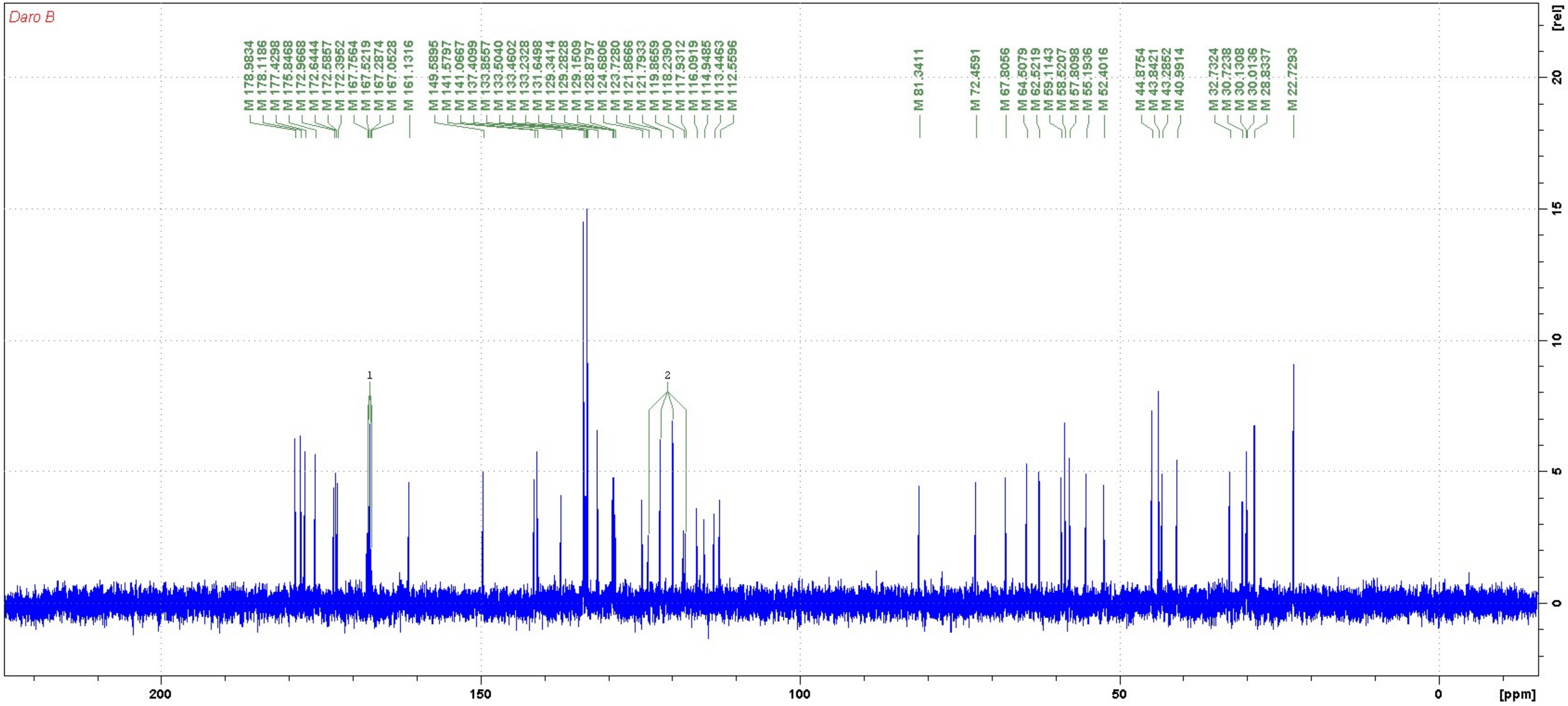

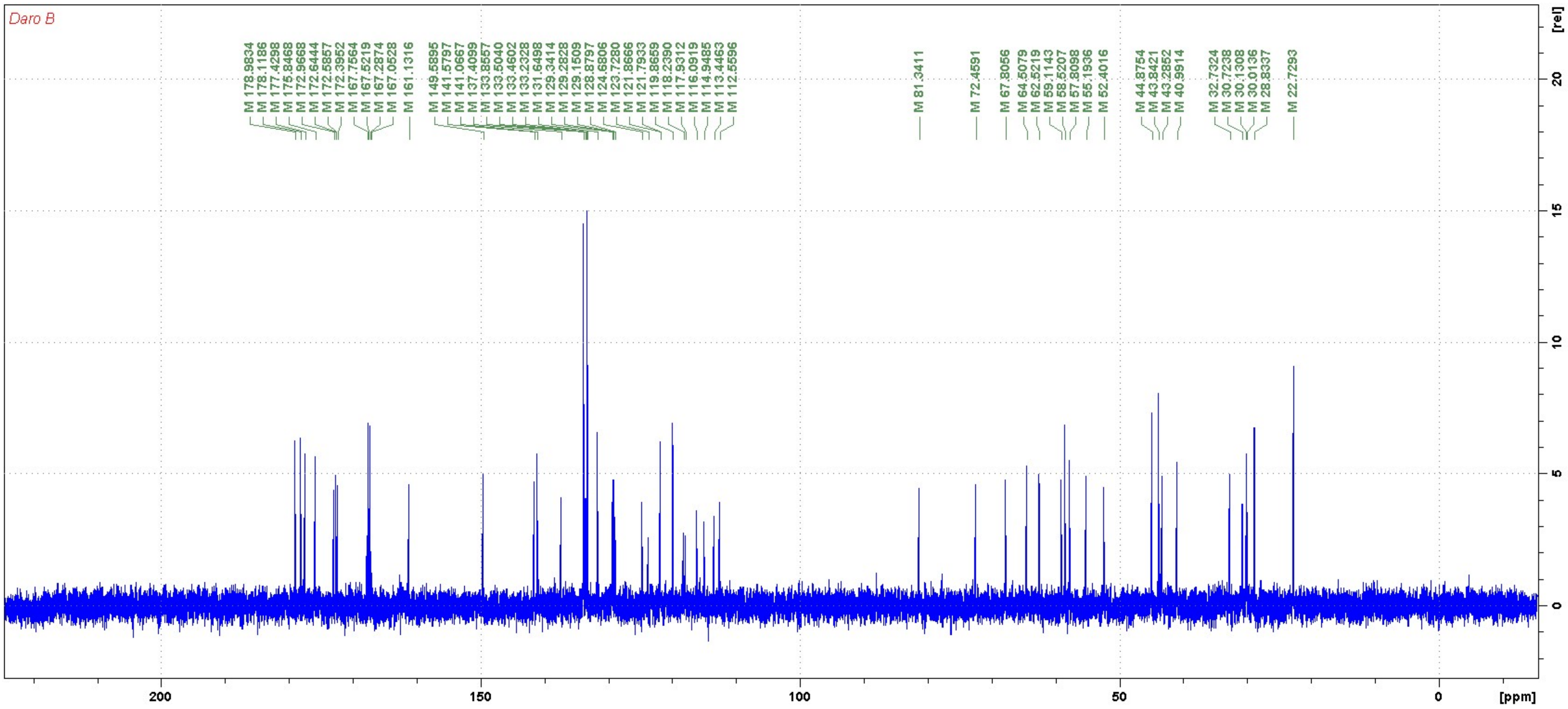

Daro B
M 178.9834
M 178.1186
M 177.4298
M 175.8468
M 172.9668
M 172.6444
M 172.5857
M 172.3952
M 167.7564
M 167.5219
M 167.2874
M 167.0528
M 161.1316
M 149.5895
M 141.5797
M 141.0667
M 137.4099
M 133.8557
M 133.5040
M 133.4602
M 133.2328
M 131.6498
M 129.3414
M 129.2828
M 129.1509
M 128.8797
M 124.6806
M 123.7280
M 121.8666
M 121.7933
M 119.8659
M 118.2390
M 117.9312
M 116.0919
M 114.9485
M 113.4463
M 112.5596
M 81.3411
M 72.4591
M 67.8056
M 64.5079
M 62.5219
M 59.1143
M 58.5207
M 57.8098
M 55.1936
M 52.4016
M 44.8754
M 43.8421
M 43.2852
M 40.9914
M 32.7324
M 30.7238
M 30.1308
M 30.0136
M 28.8337
M 22.7293
[rel]
20
15
10
5
0
200
150
100
50
0
[ppm]

Figure S5D: COSY spectrum of Darobactin B in D_2O.

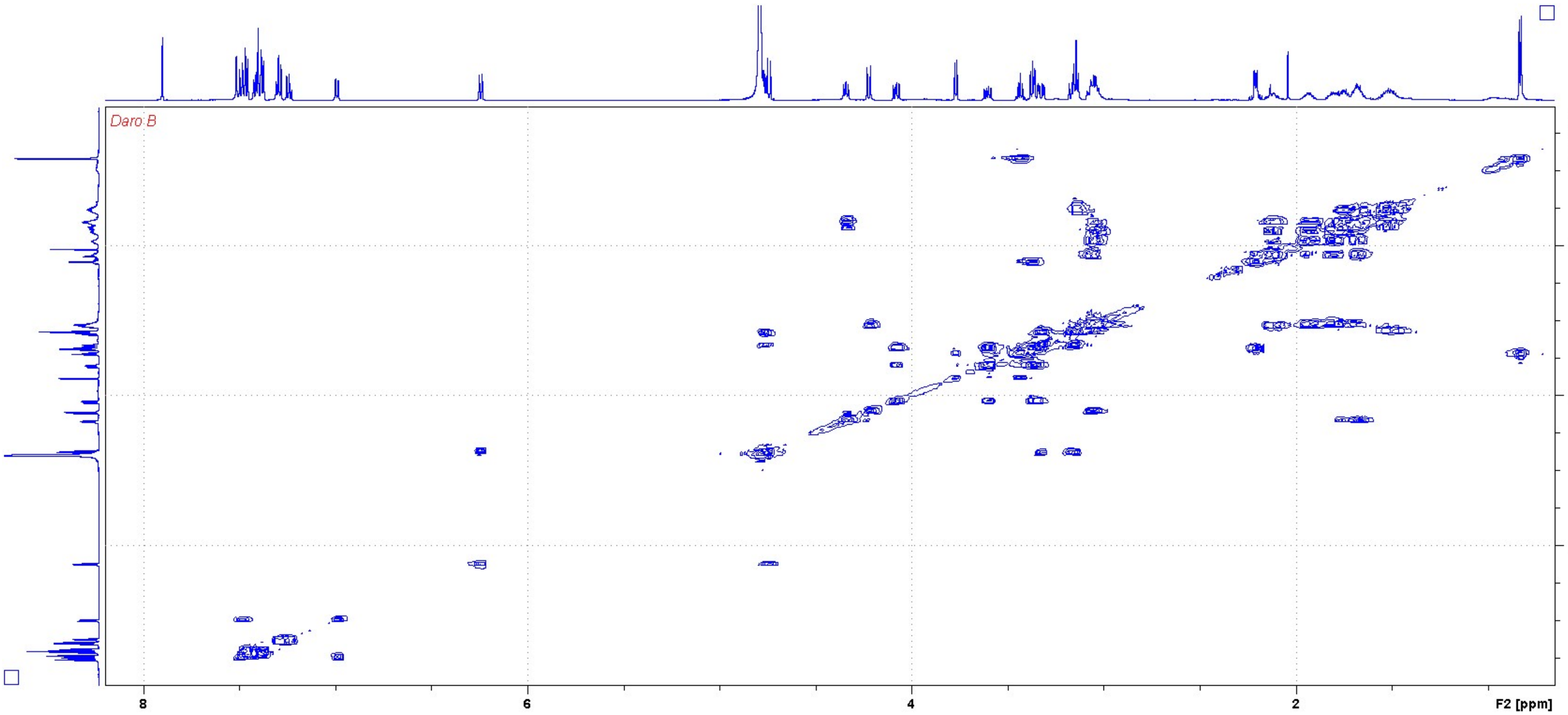

Figure S5E: TOCSY spectrum of Darobactin B in D_2O.

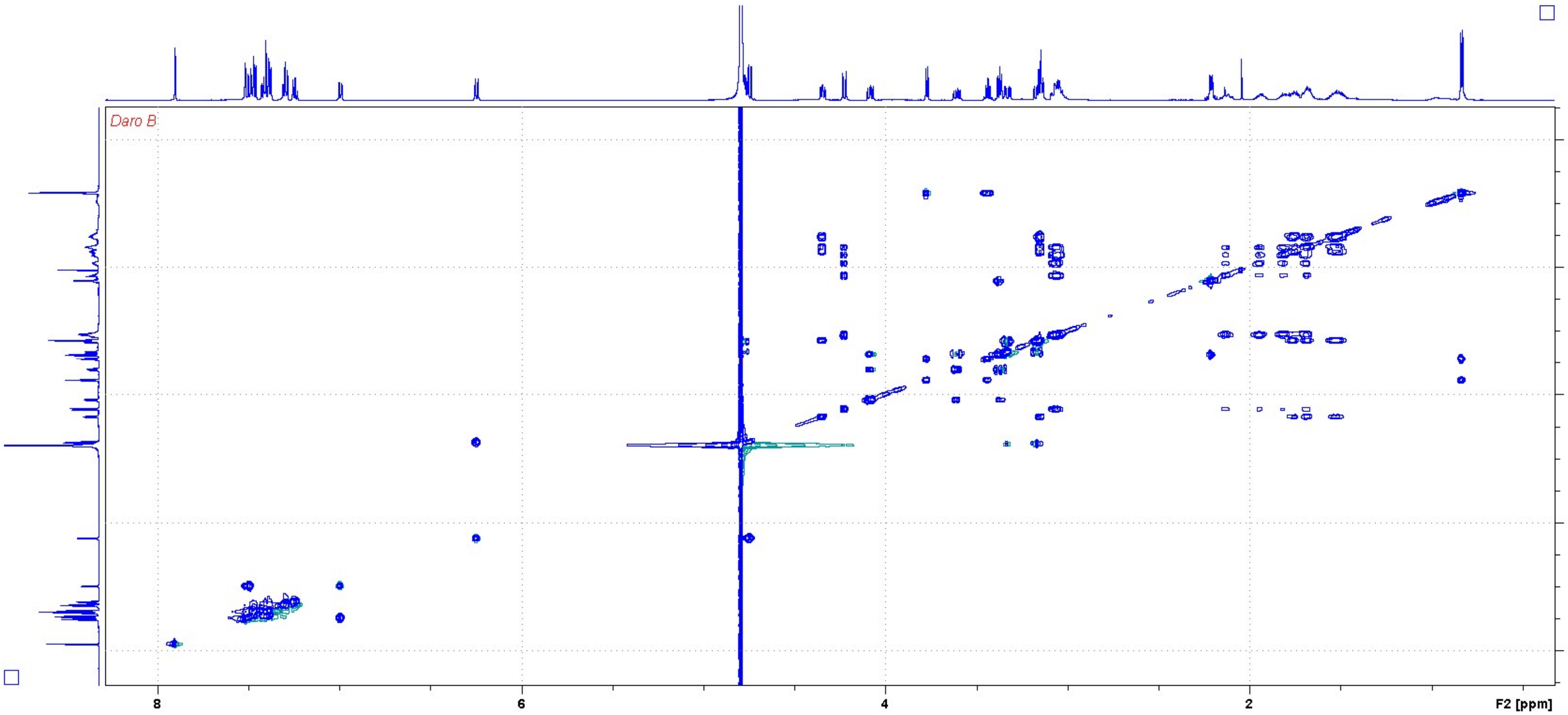

Figure S5F: HSQC spectrum of Darobactin B in D_2O.

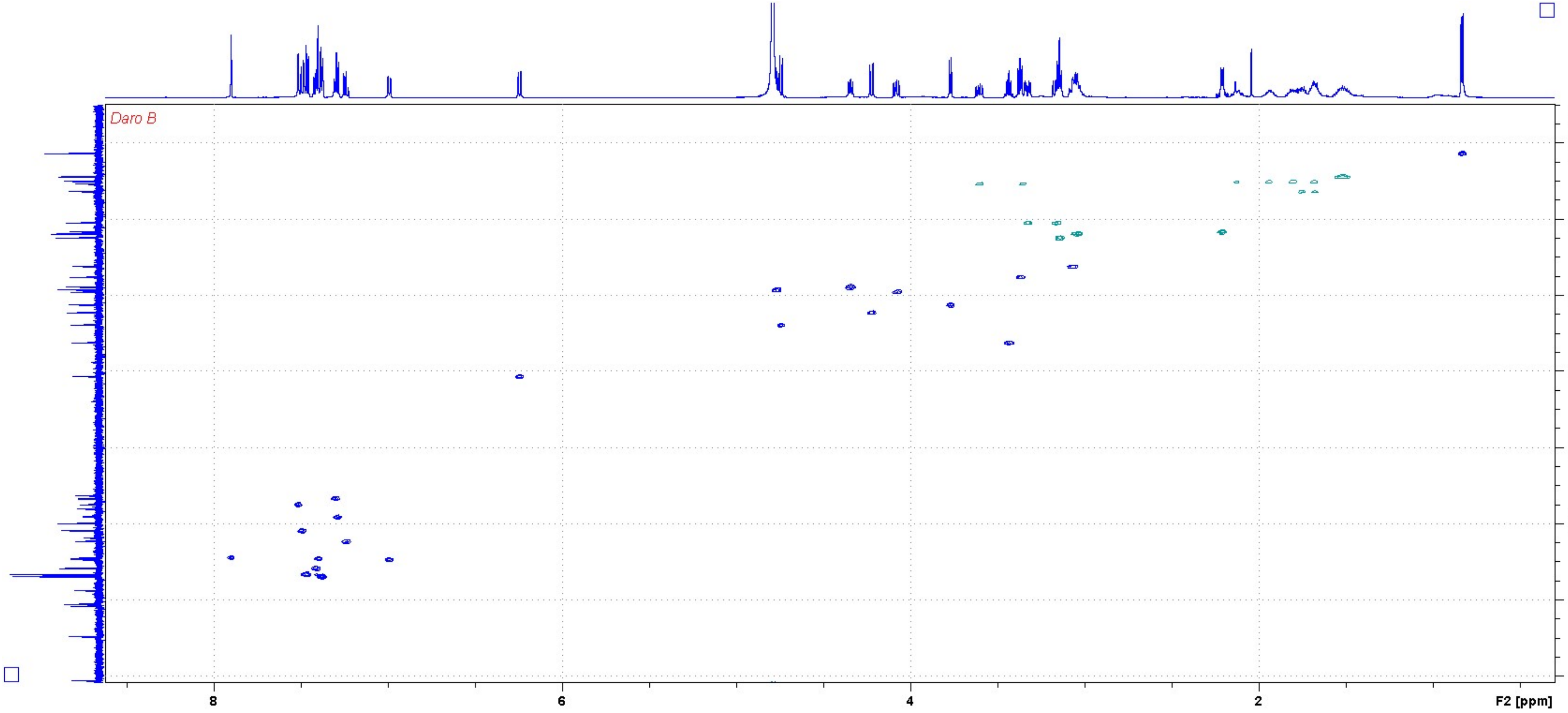

Figure S5G: HMBC spectrum of Darobactin B in D_2O. The spectrum was measured using non-uniform sampling (NUS) and H_2O suppression.

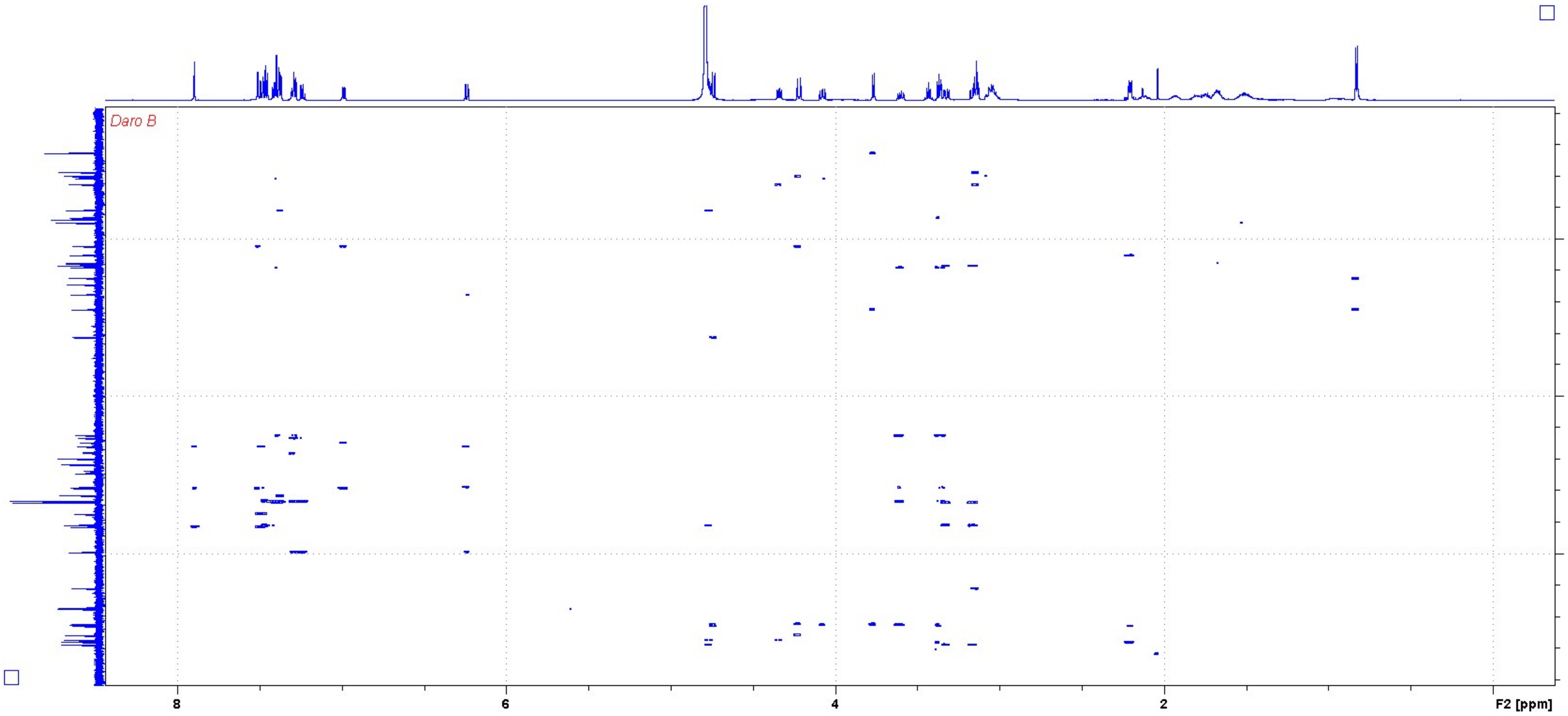

Figure S5H: NOESY spectrum of Darobactin B in D_2O.

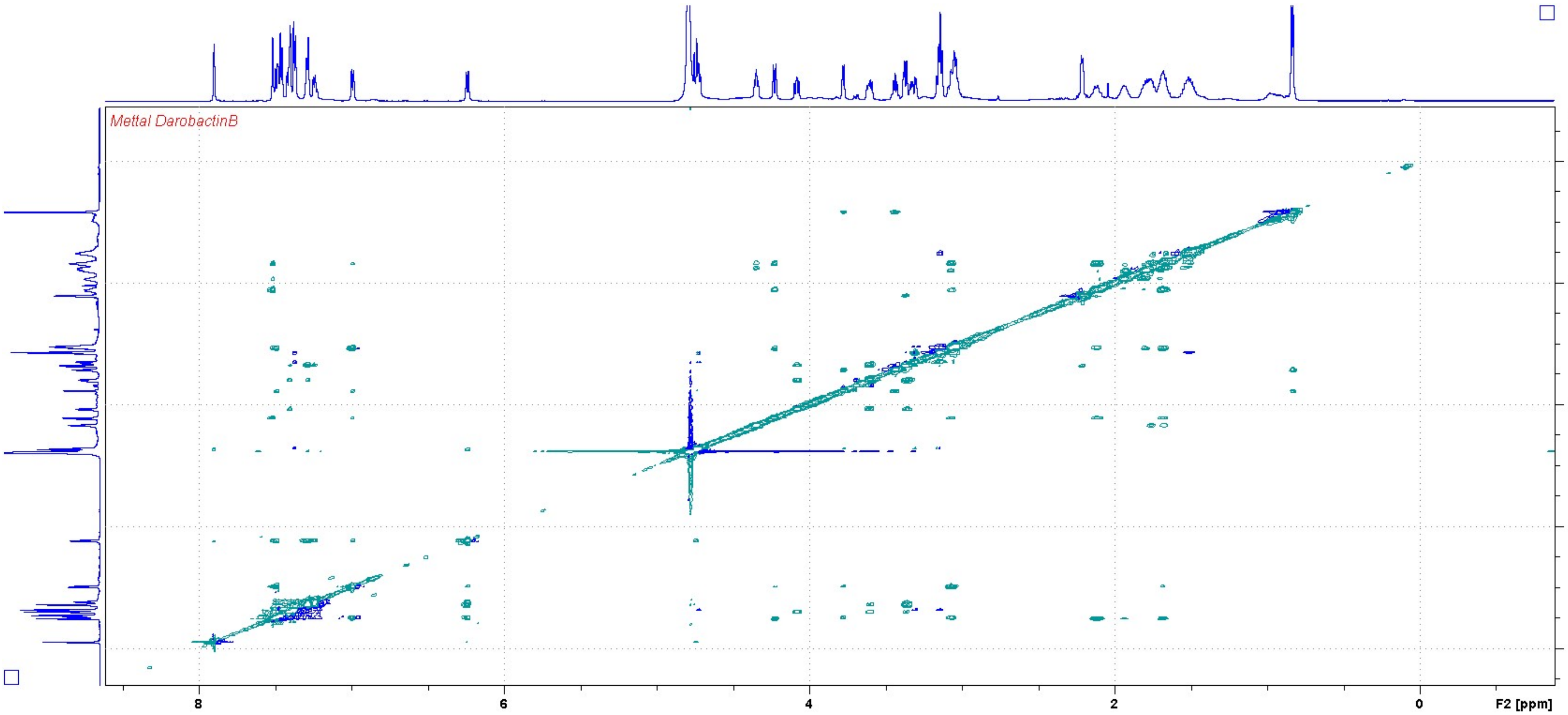

Figure S5I: ROESY spectrum of Darobactin B in D_2O.

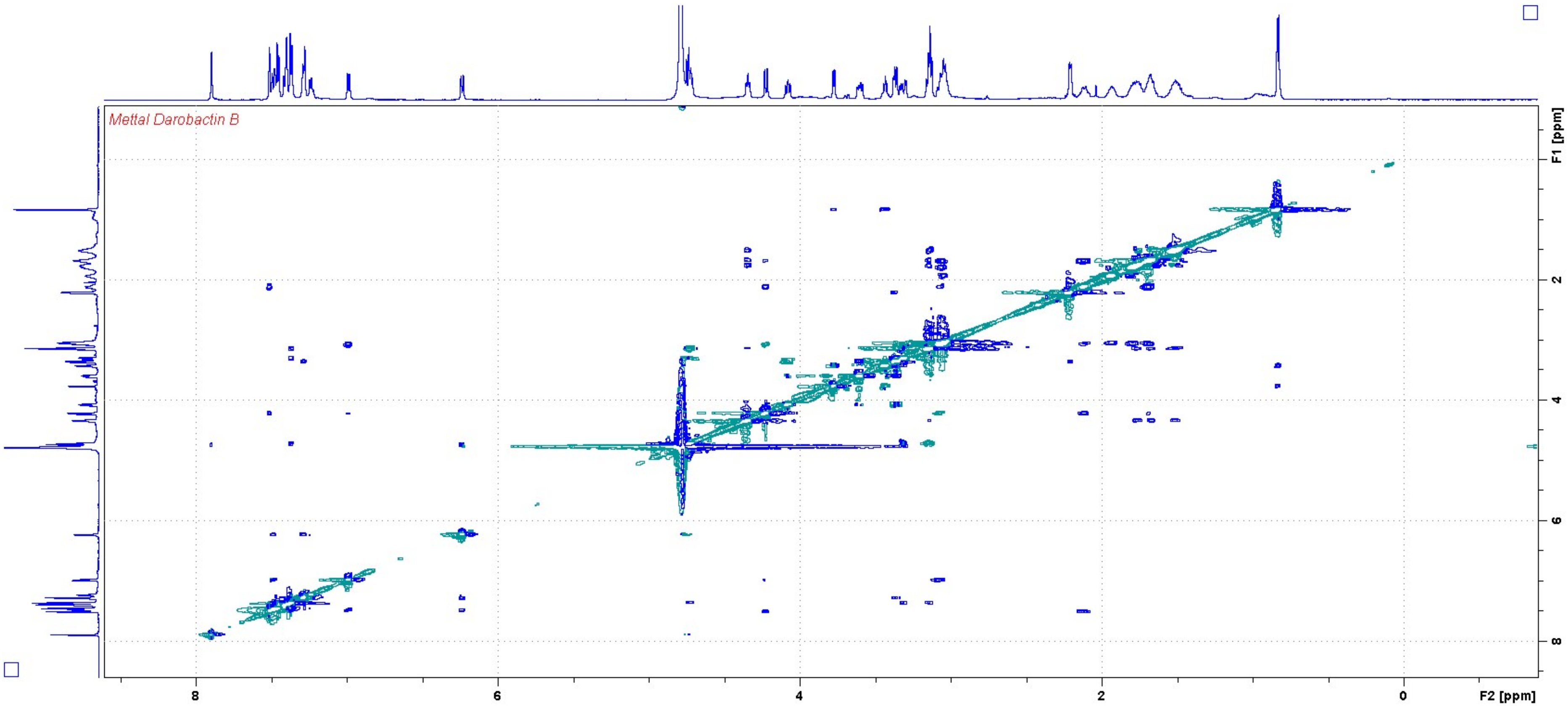

Table S2: Oligonucleotide sequences used to modify the DAR A core AAs in pNBDaromod

Primer name	Oligonucleotide sequence
DaroAf	CCTAAGATCCCTGAGATCACGGCCTGGAACTGGTCAAAAAGCTTC
DaroAr	TTTAGAAGCTTTTTGACCAGTTCCAGGCCGTGATCTCAGGGATCT
DaroBf	CCTAAGATCCCTGAGATCACGGCCTGGAACTGGACAAAAAGATTC
DaroBr	TTTAGAATCTTTTTGTCCAGTTCCAGGCCGTGATCTCAGGGATCT
DaroCf	CCTAAGATCCCTGAGATCACGGCCTGGTCATGGTCAAGATCATTC
DaroCr	TTTAGAATGATCTTGACCATGACCAGGCCGTGATCTCAGGGATCT
DaroDf	CCTAAGATCCCTGAGATCACGGCCTGGAACTGGTCAAGAAGCTTC
DaroDr	TTTAGAAGCTTCTTGACCAGTTCCAGGCCGTGATCTCAGGGATCT
DaroEf	CCTAAGATCCCTGAGATCACGGCCTGGTCATGGTCAAAGAGCTTC
DaroEr	TTTAGAAGCTCTTTGACCATGACCAGGCCGTGATCTCAGGGATCT
DaroFf	CCTAAGATCCCTGAGATCACGGCCTGGAAGTGGTCAAAGAATCTT
DaroFr	TTTAAAGATTCTTTGACCACTTCCAGGCCGTGATCTCAGGGATCT

Table S3A: Antibiogram of the clinical isolates**.** CST: colistin, AMP: ampicillin, CTX: cefotaxime, GEN: gentamycin, KAN: kanamycin, CMP: chloramphenicol, OTE: oxytetracycline, CFX: cefazidime, MPM: meropenem, IPM: imipenem, RIF: rifampicin, TET: tetracycline. Values in µg/mL. Grey boxes indicate resistance.

	Ecoli ATCC25922	*Ecoli* ATCC35218	*Ecoli* K0416	*Ecoli* MMGI1	*Ecoli* NRZ14408	*Ecoli* Survcare052
CST	<0.031	0.125	0.031	0.125	4	0.25
AMP	4	>64	>64	>64	>64	>64
CTX	<0.031	<0.031	>64	>64	>64	>64
GEN	0.5-0.25	0.25	4-2	64	>64	0.5-0.25
KAN	2	2	>64	>64	>64	1
CMP	4-2	64	64	>64	>64	4
OTE	1	2	64	>64	>64	2
CFX	0.125	0.063	64	8	32	>64
MPM	0.063	0.125	16	1	32	>64
IPM	0.125	0.125	16-8	2	8	64
RIF	4	4	8	8	>64	8
TET	0.5	1	>64	>64	64	1

Table S3B: Resistance determinants of the clinical isolates.

Isolate	Antibiotic resistance genes
NRZ14408	aac(6')Ib-cr,aadA1,aadA2,aph(3')-Ic,ARR-3,blaKPC-2,blaOXA-1,blaTEM-1B,catA1,catB3,cmlA1,dfrA18,mcr-1,mph(A),QnrB2,strA,strB,sul1,sul3,tet(A)
K0416	aac(6')Ib-cr,aacA4,aadA1,aadA5,aph(3')-Ic,blaTEM-1B,blaVIM-1,catA1,dfrA17,QnrB2,strA,strB,sul1,sul2,tet(A),tet(B)
Survcare0	mph(A),sul1,sul2,dfrA12, blaCTX-M-15,blaNDM-5,aadA2,strA,strB
MMGI1	aac(3)-IIa,aadA1,aph(3')-Ic,QnrS1,sul3,dfrA14,tet(A),floR, blaCTX-M-55,blaOXA-48,blaTEM-176

Table S4: Statistics on X-ray diffraction data and refinement of BamA-β/Darobactin B.

	**BamA-β /DarobactinB**
PDB Identifier	7P1C
Wavelength (Å)	1.00002
Resolution range (Å)	50.11 - 2.5 (2.59 - 2.5)
Space group	I 1 2 1
Unit cell	81.6 80.4 89.2
α, β, γ (°)	90 107.9 90
Unique reflections	19080 (1887)
Multiplicity	13.3 (13.0)
Completeness (%)	99.86 (100)
Mean I/sigma(I)	7.5 (1.5)
Wilson B-factor	46.8
R-merge (%)	0.278 (2.952)
Rpim (%)	0.113 (0.851)
CC1/2	0.998 (0.789)
Reflections used in Refinement	19059 (1885)
R-work	0.232 (0.301)
R-free	0.251 (0.316)
Number of atoms	3170
water	90
Protein residues	368
RMS(bonds)	0.004
RMS(angles)	1.03
Ramachandran favored (%)	96.70
Ramachandran outliers (%)	0.0
Clashscore	1.53
Average B-factor	64.0

*The values in parentheses correspond to the highest resolution shell

References

1. Anonymous. 2021. PubChem Bioassay Record for AID 1937, Source: Burnham Center for Chemical Genomics, *on* PubChem. Accessed May 5th, 2021.

2. Adams KJ, Pratt B, Bose N, Dubois LG, St John-Williams L, Perrott KM, Ky K, Kapahi P, Sharma V, MacCoss MJ, Moseley MA, Colton CA, MacLean BX, Schilling B, Thompson JW, Alzheimer's Disease Metabolomics C. 2020. Skyline for Small Molecules: A Unifying Software Package for Quantitative Metabolomics. J Proteome Res 19:1447-1458.
